# Effect of Heat Treatment on the Corrosion and Wear Behavior of Hastelloy C276 Alloy Fabricated via Laser Powder Bed Fusion

**DOI:** 10.3390/ma19112332

**Published:** 2026-06-01

**Authors:** Xiao Fang, Zitong Wang, Changqing Ye, Yang Li, Liping Zhang, Jianzhong Yang, Shulong Ye, Xin Shang, Dingning Wang, Dongyu Liu, Shukui Li, Bingwen Lu

**Affiliations:** 1 Sino-French Institute of Nuclear Engineering and Technology, Sun Yat-sen University, Zhuhai 519082, China; fangx26@mail.sysu.edu.cn; 2 Faculty of Materials Science, Shenzhen MSU-BIT University, Shenzhen 518172, China; wzt18853548096@163.com (Z.W.); 6420230005@smbu.edu.cn (L.Z.); 6420230093@smbu.edu.cn (J.Y.); 6120210001@smbu.edu.cn (S.L.); 3 Department of Materials Science and Engineering, Beijing Institute of Technology, Beijing 100081, China; 4 National-Local Joint Engineering Laboratory of Intelligent Manufacturing Oriented Automobile Die and Mould, Tianjin University of Technology and Education, Tianjin 300222, China; liyang@tute.edu.cn (Y.L.); 18834074992@163.com (D.W.); 5 School of Mechanical Engineering, Dongguan University of Technology, Dongguan 523808, China; shangxin0375@126.com (X.S.); dy20000807@126.com (D.L.); 6 State Key Laboratory of Special Materials Surface Engineering, Institute of New Materials, Guangdong Academy of Sciences, Guangzhou 510651, China; 7 Guangdong-Hong Kong Joint Laboratory of Modern Surface Engineering Technology, Guangdong Provincial Key Laboratory of Modern Surface Engineering Technology, Guangzhou 510651, China

**Keywords:** laser powder bed fusion, C276, heat treatment, corrosion behavior, friction mechanism

## Abstract

Traditional C276 alloy plates exhibit relatively poor wear resistance. Consequently, in high-wear service environments, they typically require reinforcement through additional surface coatings or wear-resistant materials. To further expand the application potential of C276 alloy in marine environments, where both corrosion resistance and wear resistance are critical, this study utilized L-PBF technology to fabricate C276 alloy specimens. The specimens were subjected to microhardness, room-temperature tensile, electrochemical corrosion, and tribological wear tests under three distinct heat treatment conditions. The results indicate that the precipitation of a Mo/W-rich μ-phase, induced by heat treatment, serves as the key factor in tailoring the material’s properties. Heat treatment was found to significantly enhance both the corrosion resistance and wear resistance of the L-PBF–fabricated C276 alloy. Specifically, the heat treatment process involving holding at 870 °C for 8 h followed by furnace cooling demonstrated the most effective strengthening effect. Under these conditions, both the microhardness and tensile strength were markedly higher than those of traditional plate specimens, thereby significantly improving the material’s damage resistance. Furthermore, the primary wear mechanisms observed in the specimens were adhesive wear and abrasive wear, accompanied by minor oxidative wear. Compared to traditional plate material, the wear rate of the heat-treated L-PBF C276 alloy was significantly reduced. This study demonstrates that appropriate heat treatment processes provide an effective pathway for tailoring the properties of L-PBF–fabricated C276 alloy components, a finding of significant importance for extending their service life and expanding their engineering applications.

## 1. Introduction

Nickel-based superalloys possess a combination of excellent mechanical properties and corrosion resistance, enabling them to withstand loads under high-temperature conditions approaching 80% of their melting point. Consequently, they are widely utilized in fields such as the chemical and nuclear industries for critical components like valves and pump bodies. During service, these components are frequently exposed to corrosive environments or even coupled tribocorrosion conditions [1,2,3,4]. C276 is a representative Ni-Cr-Mo series nickel-based alloy, primarily composed of elements such as Ni, Cr, Mo, Fe, and W. It exhibits exceptional corrosion resistance and high-temperature stability, qualities largely attributed to the solid-solution strengthening effects of its various alloying elements [5,6]. Although C276 alloy boasts superior corrosion resistance and high mechanical strength, with its tensile properties remaining relatively stable at temperatures below 850 °C, its wear resistance remains limited due to its relatively low surface hardness (230 HV–375 HV), a consequence of the high plasticity of its matrix [7,8,9]. Furthermore, traditional hot-working processes for C276 alloy face challenges such as a narrow processing temperature window and high deformation resistance [10]. Concurrently, its pronounced work-hardening tendency and high deformation resistance constrain cold-working formability, thereby leading to material wastage and increased manufacturing costs [11,12].

Additive manufacturing enables the fabrication of complex components while simultaneously achieving high material utilization efficiency [13]. Among these, laser powder bed fusion (L-PBF) is a representative metal powder bed additive manufacturing technique, also commonly referred to as selective laser melting (SLM) [14,15]. Consequently, it offers distinct advantages in terms of material conservation and manufacturing cycle reduction. The primary objective of SLM is to produce high-quality components characterized by high density, controllable surface quality, minimal residual defects, and stable mechanical properties [16,17]. Key process parameters, such as laser power, scanning speed, hatch spacing, and scanning strategy, collectively determine the microstructural characteristics and service performance of SLM-fabricated components [15,18]. Mazalov et al. [19] investigated IN718 samples fabricated via SLM and found that their creep performance along the build direction (3D printing direction) was superior to that along the scanning direction. This anisotropic creep behavior was primarily attributed to directional variations in elastic modulus and differences in grain boundary density. Tan et al. [20] demonstrated that the scanning strategy plays a pivotal role in shaping the microstructure and corrosion resistance of SLM-fabricated IN718 alloys. The samples achieved a relative density as high as 98.86%, with cellular/columnar Laves phases distributed throughout the γ-matrix, yet the alloy exhibited severe cracking following potentiodynamic polarization corrosion testing. Zhu et al. [21] successfully suppressed the formation of large-scale defects by optimizing SLM process parameters. Quantitative analysis revealed that within an optimal energy density range of 45.3 to 85.2 J/mm**^3^**, the relative density of the fabricated components consistently exceeded 99.3%. By precisely controlling secondary phases, grain morphology, grain boundary misorientation, and solidification microstructure, the defect-free laser additive manufacturing of nickel-based superalloys containing high concentrations of Al and Ti can be successfully achieved.

In mechanical engineering applications, the wear and corrosion of metals can result in significant material loss. Heat treatment and surface modification techniques represent one of the most economical and practical approaches for enhancing wear resistance [22]. Post-heat treatment processing exerts a significant influence on the microstructure and the formation of precipitate phases in C276 alloy. Deng et al. [23] fabricated C276 using a hybrid laser-arc additive manufacturing technique. Following a solution treatment at 1177 °C for 30 min followed by water quenching, the specimens underwent recrystallization, resulting in the formation of equiaxed grains accompanied by annealing twins and a reduction in texture intensity. The average grain size was further refined to 80.56 μm, and the P-phase precipitates were completely dissolved, yielding superior properties characterized by high tensile strength and exceptional elongation. Wu et al. [24] utilized an arc additive manufacturing process to deposit C276 onto P91 low-alloy creep-resistant steel. No macroscopic defects were observed in the as-deposited material. Subsequent heat treatment, involving holding at 720 °C for 2 h followed by water quenching, significantly altered the microstructures of both the C276 and P91 steel. These alterations included the promotion of grain growth, changes in grain orientation, and an increase in high-angle grain boundaries, factors critical to the enhancement of overall performance. In particular, the presence of high-angle grain boundaries within the cladding layer serves to inhibit the initiation and propagation of cracks. Qiu et al. [25] conducted heat treatments on C276 fabricated via a gas tungsten arc-arc additive manufacturing process. They found that when the material was held at 871 °C for 6 h followed by water quenching, both its strength and hardness increased significantly. However, the elongation decreased due to the formation of P-phase precipitates along the grain boundaries. Conversely, a solution treatment performed at 1177 °C for 30 min, which resulted in the partial dissolution of P-phase precipitates in the interdendritic regions and the complete dissolution of those at the grain boundaries, effectively reduced anisotropy while simultaneously enhancing both strength and plasticity.

Currently, research regarding the microstructural evolution of L-PBF–fabricated C276 alloy following heat treatment, as well as the corresponding changes in its mechanical, corrosion, and tribological properties, remains relatively limited. In contrast, current studies concerning the post-processing of additively manufactured C276 alloy primarily focus on the microstructural evolution and mechanical property variations of materials produced via processes such as Wire Arc Additive Manufacturing (WAAM), Gas Tungsten Arc Welding (GTAW), or Directed Energy Deposition (DED). Relevant studies [26] indicate that the initial microstructure of additively manufactured C276 typically exhibits pronounced directional solidification characteristics and anisotropy. These features exert a significant influence on the material’s subsequent response to heat treatment. On the other hand, studies focusing on conventionally processed or surface-modified C276 alloys [27] demonstrate that heat treatment or surface remelting can significantly alter the microstructure of the surface layer and modify its wear resistance. Furthermore, short-duration aging treatments can impact corrosion resistance by inducing the formation of Mo/W-rich precipitates and adjacent solute-depleted zones. However, systematic investigations into the synergistic changes in the corrosion and tribological behaviors of L-PBF–fabricated C276 alloy following heat treatment remain scarce, with a particular dearth of comprehensive analyses examining the interrelationship between “microstructural reconstruction,” “precipitate evolution,” and the “coupled corrosion-wear response.” Consequently, building upon existing research regarding the principles of microstructural and mechanical property control, this study focuses specifically on the synergistic corrosion and wear responses of L-PBF–fabricated C276 alloy subjected to various heat treatments, aiming to provide a foundational basis for designing optimal heat treatment protocols for this alloy in complex service environments.

## 2. Experimental Materials and Methods

### 2.1. Experimental Sample Preparation

**Figure 1:**
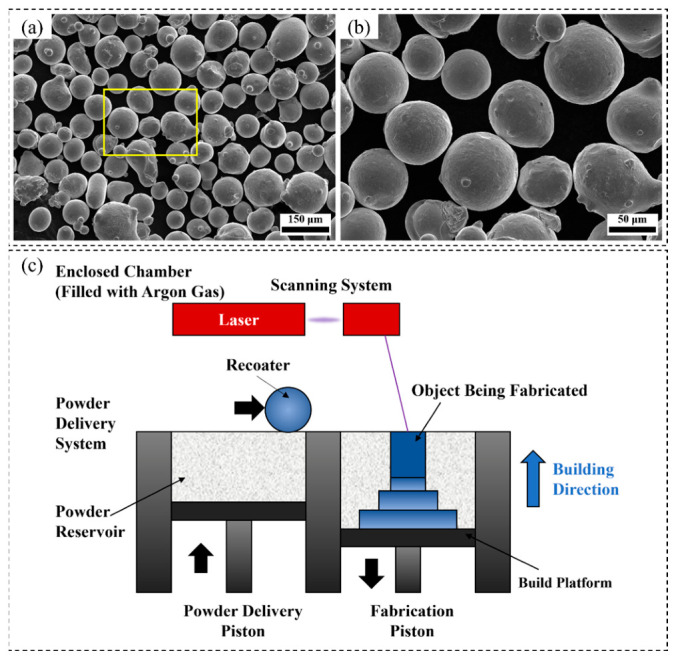
C276 alloy powder: (**a**) low-magnification SEM image, (**b**) high-magnification image, (**c**) schematic diagram of the L-PBF forming principle.

Figure 1a,b presents the scanning electron microscope (SEM) morphology of the C276 alloy powder. The powder consists predominantly of spherical particles, indicating excellent flowability and powder-spreading characteristics. The powder exhibits a relatively broad particle size distribution, estimated statistically based on the SEM images in Figure 1, with an average particle size of approximately 40 μm and a primary concentration within the 20–60 μm range. This moderate particle size distribution facilitates improved powder-spreading uniformity and melt pool stability, thereby enabling the fabrication of L-PBF components with high density. In this study, an EOS M290 system was utilized for the L-PBF fabrication process. The fundamental principle of this process is illustrated in Figure 1c. Bulk C276 specimens with dimensions of 50 mm × 10 mm × 10 mm were successfully fabricated. Table 1 lists the chemical composition of the powder. Based on extensive preliminary process trials and optimization of the forming quality, the combination of L-PBF process parameters selected in this study was validated to yield low porosity, high density, and sound metallurgical bonding. As this parameter window consistently produces formed samples characterized by high density and low porosity, this paper does not focus on the influence of forming parameters on densification behavior, nor does it undertake a comparative analysis of different L-PBF parameter combinations. Instead, it centers on the role of heat treatment in tailoring the microstructure and comprehensive properties. The specific fabrication parameters employed were as follows: layer thickness of 40 μm, laser power of 285 W, scanning speed of 960 mm/s, and hatch spacing of 0.11 mm. All performance test specimens were sectioned from the same batch of L-PBF–fabricated blocks to ensure comparability among the various test results. Additionally, conventionally rolled C276 sheet material was included as a reference sample for comparison.

Drawing upon extensive preliminary explorations of various heat treatment regimens, the three specific processes systematically selected for this study are representative in terms of their influence on mechanical strength and effectively span the critical parameter ranges that govern the resulting microstructure and mechanical properties. Table 2 shows the heat treatment parameters of the samples. Previous studies have demonstrated that Alloy C276 is prone to grain boundary precipitation within the temperature range of 649 to 1093 °C, leading to the formation of P phase, μ phase, or Mo/W-rich precipitates, which significantly impact the alloy’s mechanical properties and corrosion resistance. In particular, prolonged aging near 800 °C promotes the formation of Mo/W-rich carbides or Topologically Close Packed (TCP)-type precipitates at grain boundaries, accompanied by the depletion of Mo and W in the adjacent regions. Consequently, this study selects 720 °C and 870 °C as representative low to intermediate heat treatment temperatures to investigate the respective effects of low temperature aging and intermediate temperature heat treatment on precipitation behavior, grain boundary stability, and overall performance. Specifically, the fine precipitate phases formed during the secondary aging process, along with their pinning effect at interfaces, can reduce grain boundary mobility. Furthermore, holding at low temperatures helps stabilize the substructure established during the preceding heat treatment, thereby inhibiting further grain growth. Concurrently, HT3 was subjected to a subsequent low-temperature aging treatment at 620 °C following the 870 °C treatment, to evaluate the effects of this secondary aging on the dispersed distribution of precipitate phases, substructure stabilization, and the inhibition of grain growth. Upon completion of the fabrication process, the specimens underwent three distinct heat treatments involving a heating rate of 10 °C/min. The specific parameters for each treatment are detailed in Table 2. Subsequently, the specimens were sequentially ground using silicon carbide papers ranging from 180 to 2500 grit, followed by fine polishing using a 0.03 μm SiO_2_ suspension. Subsequently, electropolishing was performed for 30 s at 26 V using an electrolyte consisting of 70% C_2_H_5_OH, 10% H_2_SO_4_, and 20% H_2_O. Finally, the sample was rinsed with distilled water and dried with hot air.

### 2.2. Microstructural Analysis

After cutting and cold-mounting, the specimens were ultrasonically cleaned in anhydrous ethanol, then ground and polished to a mirror-like finish. The polished samples were etched using aqua regia (HCl:HNO_3_ = 3:1, by volume) for several to tens of seconds, with the specific duration adjusted based on the clarity of the revealed microstructure [6]. Microstructural observations were performed using a ZEISS Axio Imager.M2m 3D optical microscope (OM) (ZEISS, Oberkochen, Germany). A Phenom Nano G2 scanning electron microscope (SEM) (FEI, Phenom Nano G2, Eindhoven, Netherlands) was employed to observe the morphology of precipitates formed during heat treatment, and its accompanying energy-dispersive X-ray spectrometer (EDS) was utilized for elemental analysis. Prior to SEM/EDS analysis, the specimen was secured to the sample stage using conductive adhesive, ensuring that the overall height of the sample remained under 6 mm. It was then placed into the SEM vacuum chamber and evacuated to a high-vacuum state. Under an accelerating voltage of 15 kV, the microstructure morphology was observed using the secondary electron mode, and EDS elemental area scanning analysis was subsequently performed under identical conditions. Electron backscatter diffraction (EBSD) analysis was conducted using a Sigma500 system (ZEISS, Germany) with a step size of 0.8 μm to evaluate specific regions and characterize the grain orientation, size, and shape of the various samples. Based on preliminary microstructural characterization, including EBSD analysis, of the XZ and XY cross-sections, it was determined that the XZ plane exhibited a lower dislocation density and superior crystallographic characteristics. Consequently, this study selects the XZ plane as the primary testing cross-section. The rationale behind choosing a horizontal orientation is to align the testing plane with the loading and friction directions, thereby minimizing the influence of build orientation variations on the results and highlighting the specific effects of heat treatment variables. It should be noted that L-PBF–fabricated C276 alloy may exhibit significant orientation-dependent effects. Vertically oriented specimens are typically more susceptible to the continuous growth of columnar grains along the build direction and the influence of interlayer fusion boundaries, whereas specimens with an inclined orientation exhibit microstructural characteristics that represent a coupling of both the scanning and build directions. Consequently, different orientations may lead to variations in grain morphology, texture intensity, balance between strength and ductility, and wear behavior. To ensure comparability and to focus specifically on the effects of heat treatment, all subsequent investigations into mechanical, electrochemical, and tribological properties were conducted exclusively on the XZ plane. The microstructural evolution and property variations associated with different build orientations will be systematically analyzed in future studies.

### 2.3. Mechanical Properties

**Figure 2:**
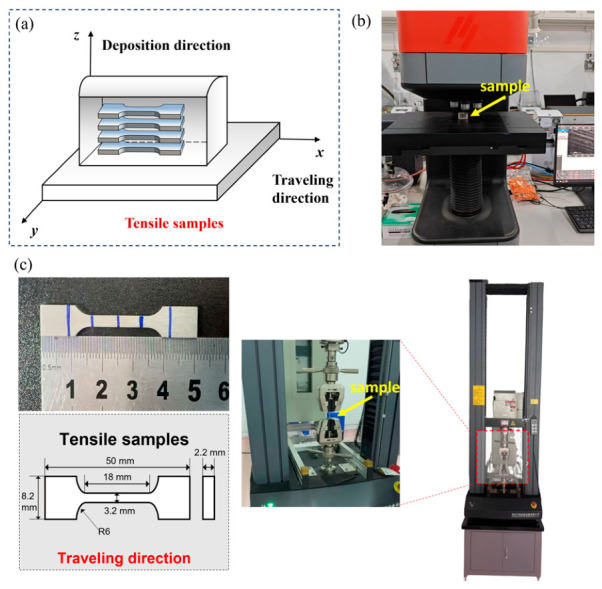
Schematic diagrams of specimen sampling methods and hardness and tensile testing equipment. (**a**) Schematic of sampling for various test specimens after L-PBF fabrication. (**b**) Microhardness testing equipment and specimen mounting configuration. (**c**) Photograph and dimensional schematic of the tensile specimen, along with the tensile testing equipment and specimen mounting configuration.

Figure 2 presents a schematic diagram illustrating the specimen sampling method, as well as the hardness and tensile testing equipment. Hardness testing is one of the key indicators for evaluating the mechanical properties of metals [28]. The hardness of the specimens was measured using a DURAMIN-40A3 microhardness tester (STRUERS, Ballerup, Denmark), with a load of 0.5 kg and a dwell time of 10 s. Ten test points were selected for each specimen, with a spacing of 3 mm between points. Based on the design principles for small sized plate type tensile specimens of metallic materials, the L-PBF fabricated specimens were machined into small sized plate type tensile specimens. Their room-temperature tensile properties were evaluated using a computer-controlled electronic universal testing machine, model ETM 304C (Wance, Shenzhen, China), at a tensile rate of 0.5 mm/min. At least three room-temperature tensile tests were conducted for each condition, and the results are presented as the mean and standard deviation.

### 2.4. Electrochemical Analysis

To simulate a marine service environment, electrochemical tests were conducted at room temperature in a 3.5 wt.% NaCl solution using a CHI760E electrochemical workstation (Chenhua Instruments, Shanghai, China). Table 3 shows the electrochemical corrosion test parameters. A conventional three-electrode system was employed for the electrochemical measurements, utilizing a saturated KCl Hg/HgCl_2_ electrode as the reference electrode, a platinum sheet as the counter electrode, and the specimen as the working electrode. Prior to formal testing, the specimen was immersed in the electrolyte, and the open-circuit potential (OCP) was monitored for 3600 s to allow the system to reach a steady state. Subsequently, potentiodynamic polarization tests were performed within the potential range of −1 to 1 V at a scan rate of 1 mV·s^−1^. It should be noted that in the experiment, the anodic branch does not exhibit a stable Tafel linear region in the vicinity of *E_corr_*. Rather, it is influenced by the formation of a surface passivation film, localized film breakdown, and subsequent repassivation processes. Consequently, the anodic branch was not utilized for Tafel extrapolation in this study. Instead, the corrosion current density (*I_corr_*) was determined solely by extrapolating the linear region of the cathodic Tafel branch to Ecorr [29,30]. *I_corr_* was treated as a fitted parameter and was not obtained by directly reading the lowest current point on the polarization curve. All polarization tests were repeated at least three times, and the data were averaged. Electrochemical impedance spectroscopy (EIS) tests were conducted over a frequency range of 10^5^ to 10^−2^ Hz, with a perturbation voltage amplitude of 5 mV. To ensure the reliability of the results, each set of experiments was repeated at least three times.The EIS data were fitted using a nonlinear least-squares method based on an equivalent circuit model.

### 2.5. Friction and Wear Test

**Figure 3:**
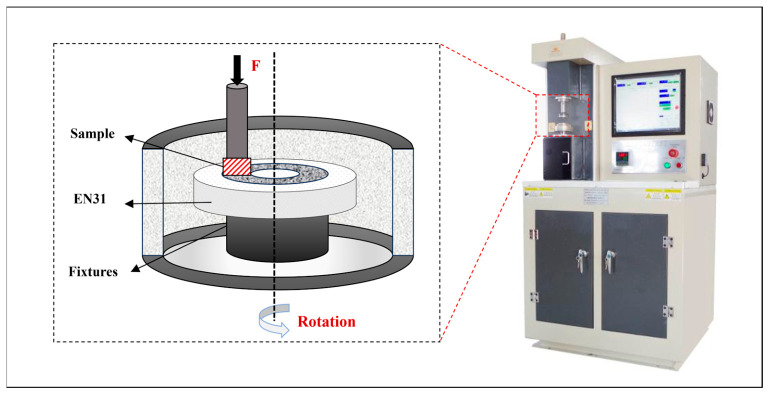
Schematic diagram of friction and wear.

Friction and wear tests were conducted under dry friction conditions at room temperature, as shown in Figure 3. The counter-disc material consisted of hardened EN31 steel (55–60 HRC) [31], the chemical composition of which is presented in Table 4. The dimensions of the specimens were 10 mm × 10 mm × 2 mm. The applied load was 60 N, and the rotational speeds were set to 30 and 60 r/min, respectively. Specific parameters are detailed in Table 5.

The aforementioned settings for load, rotational speed, and test duration were determined based on a comprehensive assessment of preliminary experiments and the relevant literature concerning similar dry sliding friction conditions. This approach aimed to elicit stable and discernible wear behavior under ambient temperature conditions while simultaneously preventing surface failure of the specimens under excessively high loads or prolonged friction durations. Prior to and following the tests, the specimens were polished and cleaned with acetone. In this study, no separate, dedicated run-in tests were conducted. Instead, the initial phase of the friction curve was treated as the natural run-in process of the material-counterpart system. Upon completion of the tests, mass loss was determined using an electronic balance, while friction force and friction coefficient were recorded in real time via force sensors integrated into the testing equipment. A Phenom Nano G2 scanning electron microscope (SEM) (FEI, Phenom Nano G2, Eindhoven, The Netherlands) was utilized to observe the morphology of the wear tracks and wear debris, as well as their elemental distribution. A DURAMIN-40A3 microhardness tester was employed to measure the microhardness of the specimens after wear testing. The profiles of the wear tracks and the surface roughness were measured using an Alpha-Step D-600 2D profilometer (KLA, Milpitas, USA) and an OLS 50,003 laser scanning confocal microscope (Olympus Corporation, Tokyo, Japan), with a scanning speed of 0.5 mm/s and a scanning length of 3 mm. The wear rate was calculated based on the mass loss, applied load, and sliding distance. The calculation formula is as follows [32,33]:

$$W=\frac{M}{F\cdot S\cdot A}$$

In this equation, *M* represents the wear volume (g), *F* is the test load, *S* is the total sliding distance (m), and *A* is the effective friction area (m^2^). The unit of *W* is g·N^−1^·m^−3^. Furthermore, the “30 + 60 r/min, 1 h” entry in Table 5 corresponds to the segmented variable-speed test condition, which is used to evaluate the overall response of the friction interface under varying rotational speeds. Conversely, the “30 r/min, 0.5 h” and “60 r/min, 0.5 h” entries are utilized for comparative wear analysis under constant-speed conditions.

## 3. Experimental Results and Analysis

### 3.1. Microstructural Analysis

**Figure 4:**
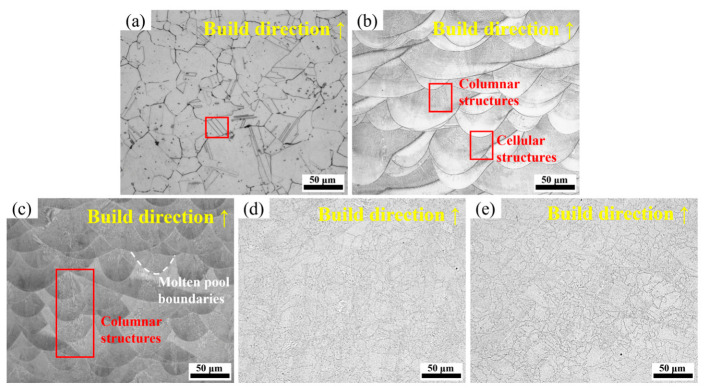
OM microstructures of the XZ cross-sections of C276 alloy in different states. The arrows indicate the build direction: (**a**) as-rolled, (**b**) as-printed, (**c**) HT1, (**d**) HT2, and (**e**) HT3.

Figure 4 presents the optical microscope (OM) microstructure morphologies of C276 alloy in different states. The conventional plate sample (Figure 4a) exhibits a typical equiaxed grain structure, accompanied by distinct annealing twins, indicating that it has undergone extensive plastic deformation followed by a recrystallization process. In contrast, the as-deposited sample (Figure 4b) displays characteristic fish-scale-like melt pool boundaries, featuring a coexistence of columnar and cellular grain structures that reflect prominent characteristics of rapid directional solidification. This specific microstructure morphology stems from the high temperature gradients and cooling rates inherent to the L-PBF process. The melt pools undergo epitaxial growth aligned with the direction of heat flow, resulting in a non-equilibrium solidification structure dominated by cellular and columnar grains, while the repeated remelting between adjacent tracks and layers further preserves the distinct melt pool boundaries [15]. No significant pores or cracks are visible in Figure 4b, indicating that, under the current processing parameters, the sample possesses a high degree of fabrication density.

Following heat treatment, the as-deposited microstructure undergoes significant evolution. In sample HT1 (Figure 4c), the contours of the melt pools remain visible, indicating that the dual-aging treatment at 720 °C was insufficient to completely eliminate the inherited as-deposited microstructure. However, fine, relatively uniform precipitate phases have emerged both within the grains and in the vicinity of the grain boundaries, suggesting that the low-temperature, long-duration aging treatment facilitated solute atom diffusion and localized precipitation while simultaneously exerting a stabilizing effect on the original substructure. In sample HT2 (Figure 4d), the original melt pool boundaries are significantly attenuated, and the microstructure as a whole tends toward greater uniformity. Sample HT3 (Figure 4e) likewise exhibits a high degree of microstructural uniformity yet features a finer grain boundary network. This indicates that the application of a secondary aging treatment, following the primary treatment at 870 °C, helps to inhibit further grain growth and promotes the dispersed distribution of fine precipitate phases.

**Figure 5:**
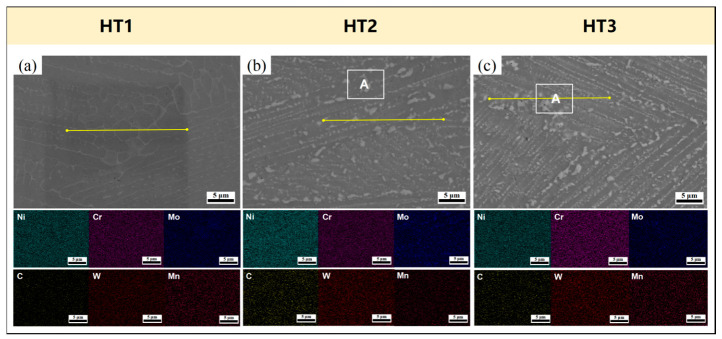
SEM morphology and EDS point analysis of precipitate phases in L-PBF C276 alloy after heat treatment: (**a**) HT1, (**b**) HT2, and (**c**) HT3.

**Figure 6:**
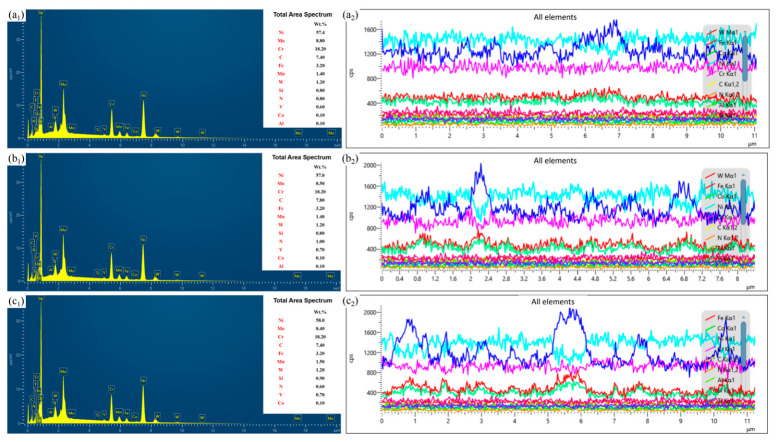
EDS spectra and line scan analysis of precipitate phases in L-PBF C276 alloy after heat treatment: (**a_1_**–**c_1_**) EDS spectra for HT1, HT2, and HT3, (**a_2_**–**c_2_**) EDS elemental line scan results acquired along the yellow line shown in Figure 5.

To further analyze the microstructural characteristics, the elemental distribution of the aged alloy was characterized using SEM and EDS, as shown in Figure 5 and Figure 6. Following heat treatments HT2 and HT3, a large number of dispersively distributed bright-white particles precipitated along the original cellular boundaries and high-angle grain boundaries. These particles predominantly exhibited spherical or short rod-like morphologies, with an average size of approximately 100 nm.

Based on the EDS point-scan data (Table 6), these white particles exhibit significant enrichment in Mo and W, accompanied by a relative decrease in Ni and Cr content. Notably, the Si content within the precipitate phase remains at a low level. Considering its compositional characteristics, specifically enrichment in Mo/W and depletion in Ni/Cr, and referencing the literature reports regarding TCP phase precipitation in C276 alloy, this precipitate phase can be identified as a Mo/W-rich μ phase. EDS line-scan results confirm that the compositional profiles of samples HT2 and HT3 display significant fluctuations between peaks and valleys, indicating that the precipitation of the μ-phase has driven a redistribution of the local chemical composition. This effect is particularly pronounced in sample HT3, where the introduction of a secondary aging treatment further intensified the enrichment of Mo and W atoms within the precipitated phase. This high-temperature aging-induced precipitation of the μ-phase not only alters the substructural morphology of both grain boundaries and grain interiors but also significantly modulates the thermodynamic state of the matrix, thereby establishing the microstructural foundation for the subsequent evolution of mechanical and electrochemical behaviors.

**Figure 7:**
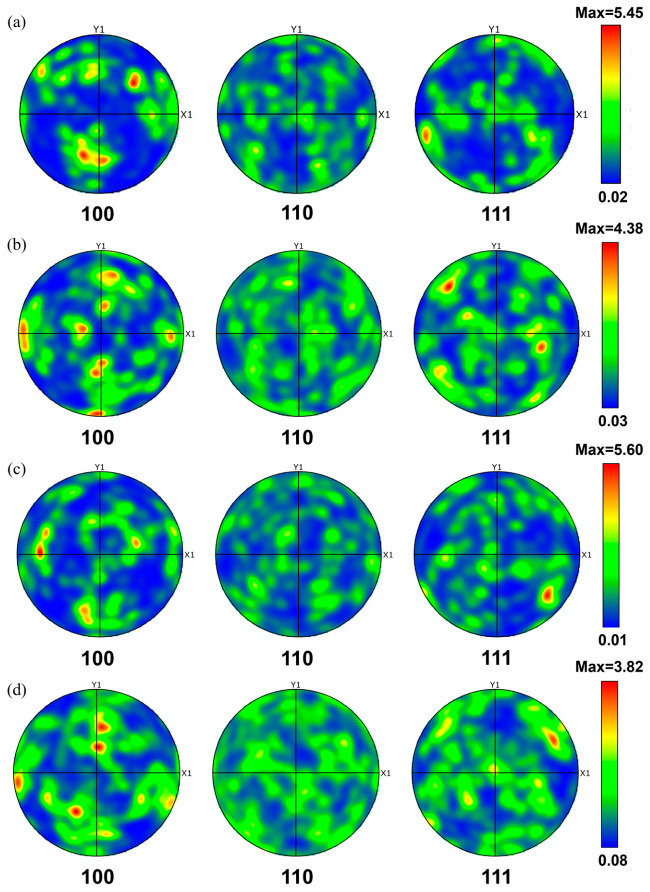
Pole figures of the XZ cross-sections of L-PBF C276 alloy in different states: (**a**) as-printed, (**b**) HT1, (**c**) HT2, and (**d**) HT3.

EBSD pole figures further revealed the influence of heat treatment on the texture evolution of the L-PBF C276 alloy. As shown in Figure 7, the as-deposited sample exhibited distinct characteristics of orientation clustering on the XZ cross-section. Localized orientation concentrations were observable in its (100), (110), and (111) pole figures, with a maximum pole density of 5.45. This indicates that the epitaxial growth occurring along the direction of the thermal gradient during the rapid solidification inherent to the L-PBF process allowed the microstructure to retain certain inherited characteristics of directional solidification. Following heat treatment, the samples retained a degree of preferred orientation, yet both the texture intensity and orientation distribution underwent significant changes. The maximum pole density of the HT1 sample decreased to 4.38, suggesting that following the two-stage aging treatment at 720 °C + 620 °C, processes such as recovery, precipitation, and localized microstructural adjustments attenuated the degree of orientation concentration present in the as-deposited state. Conversely, the maximum pole density of the HT2 sample increased to 5.60, slightly higher than that of the as-deposited state, indicating that during the single-stage heat treatment at 870 °C, certain grains underwent orientation selective growth, thereby resulting in a localized enhancement of the texture. In contrast, the maximum pole density of the HT3 sample dropped to 3.82, the lowest among all heat treated states, demonstrating that the two-stage heat treatment at 870 °C + 620 °C was most effective in promoting grain orientation dispersion and microstructural homogenization, thereby weakening the texture inherited from the as-deposited state. Overall, while heat treatment did not completely eliminate the inherited orientation characteristics formed during the L-PBF process, it effectively modulated texture intensity through mechanisms such as recovery, recrystallization, precipitation pinning, and selective grain growth. Among the treatments examined, HT3 demonstrated the most pronounced effect in terms of texture weakening and orientation homogenization.

**Figure 8:**
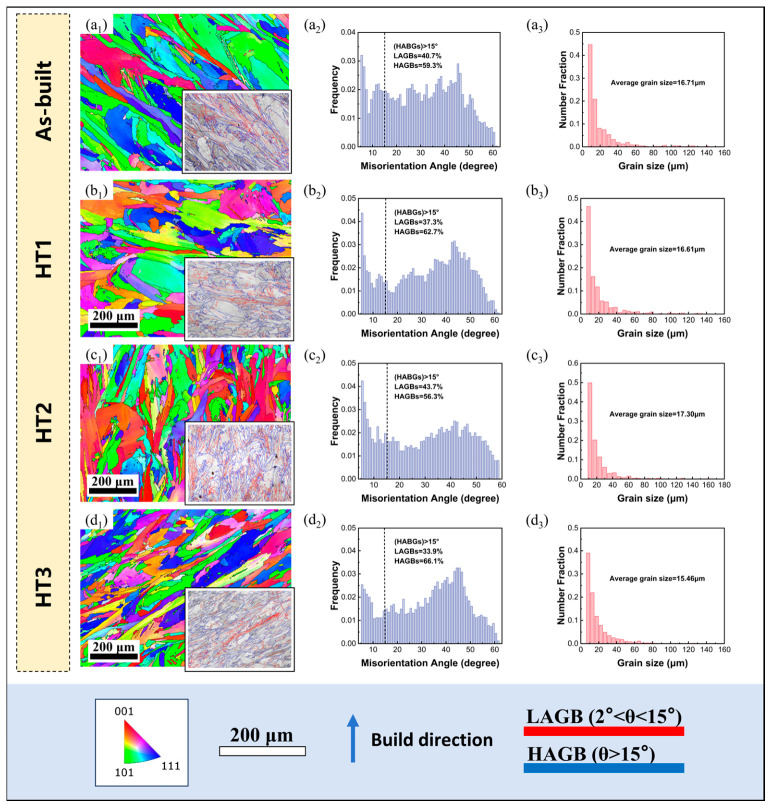
EBSD analysis of L-PBF C276 samples after heat treatment: (**a_1_**–**d_1_**) IPF maps, (**a_2_**–**d_2_**) statistical maps superimposing high- and low-angle grain boundaries, and (**a_3_**–**d_3_**) grain size distribution histograms.

As evident from the Inverse Pole Figure (IPF) orientation maps (Figure 8(a_1_–d_1_)), the as-deposited, HT1, HT2, and HT3 samples all exhibit grain characteristics elongated along the build direction. This indicates that the directional growth structures formed during the rapid solidification process of L-PBF were not completely eliminated following heat treatment. Notably, the as-deposited sample displays a distinct banded orientation distribution and continuous regions of similarly colored grains, suggesting that the as-deposited microstructure retains strong characteristics of epitaxial growth and orientation inheritance. In sample HT1, regions of continuously distributed, similarly colored grains are relatively large, and the clustering of grain orientations is quite pronounced. This indicates that following the dual-aging treatment at 720 °C, the original preferred orientation, formed through epitaxial growth during the deposition process, was largely retained, and the degree of texture weakening was limited. In sample HT2, the intermingled distribution of grains with varying colors is more apparent, and the dispersion of grain orientations increased. This suggests that the isothermal holding treatment at 870 °C facilitated grain boundary migration and orientation rearrangement, resulting in a significant weakening of the original directional solidification texture. Although sample HT3 still exhibits banded grains distributed along the build direction, its overall color distribution is more uniform than that of HT1. This indicates that the introduction of a secondary aging treatment, following the high-temperature microstructure modification, further enhanced the dispersion of grain orientations and led to a continued weakening of the texture.

Based on the statistical analysis of grain boundary types (Figure 8(a_2_–d_2_)), the fractions of low-angle grain boundaries (LAGBs, 2° < θ < 15°) in the as-deposited, HT1, HT2, and HT3 samples were 59.3%, 37.3%, 43.7%, and 33.9%, respectively. The corresponding fractions of high-angle grain boundaries (HAGBs, θ > 15°) were 40.7%, 62.7%, 56.3%, and 66.1%. The as-deposited sample exhibited the highest proportion of LAGBs, indicating that the as-deposited microstructure retained a significant number of subgrain boundaries and dislocation substructures resulting from rapid solidification, thermal cycling, and residual stresses. Following heat treatment, the proportion of LAGBs generally decreased while that of HAGBs increased significantly, suggesting that the heat treatment promoted recovery, grain boundary migration, and partial recrystallization processes, thereby attenuating the substructural characteristics of the as-deposited state. Notably, the HT2 sample displayed the highest proportion of LAGBs, implying that it still retained a relatively large number of subgrain boundaries and deformation substructures internally. This reflects that, following the high-temperature treatment, recovery was substantial, yet recrystallization had not reached full completion. Conversely, the HT3 sample exhibited the highest proportion of HAGBs, indicating a more advanced degree of grain boundary transformation and a microstructure that more closely approximates a stable, recrystallized or reconstructed state. Since high-angle grain boundaries are conducive to attenuating the strong texture and the inherited characteristics of directional epitaxial growth typical of the as-deposited state, the HT3 sample demonstrates a distinct advantage in terms of microstructure homogenization. In contrast, the retention of a higher proportion of low angle grain boundaries in the HT2 sample implies that its internal substructural features and localized stored strain remain more pronounced.

EBSD results further revealed the influence of heat treatment on grain characteristics and grain boundary structures (Figure 8(a_3_–d_3_)). According to the initial statistical analysis, the average grain size of the as-deposited sample was 16.71 μm. Following heat treatment, the average grain sizes for HT1, HT2, and HT3 were 16.61 μm, 17.30 μm, and 15.46 μm, respectively. These results indicate that HT1 is nearly comparable to the as-deposited state, suggesting that its microstructural evolution is primarily manifested through changes in precipitation and defect states rather than through significant grain refinement. The average grain size of HT2 increased to 17.30 μm, signifying that at 870 °C, grain boundary migration was active and grain growth became the dominant process. HT3 exhibited the lowest average grain size, approximately 7.5% smaller than that of the as-deposited state, demonstrating that subjecting the material to a secondary aging treatment following high-temperature microstructural reconstruction helps to suppress further grain growth and enhance microstructural uniformity.

**Figure 9:**
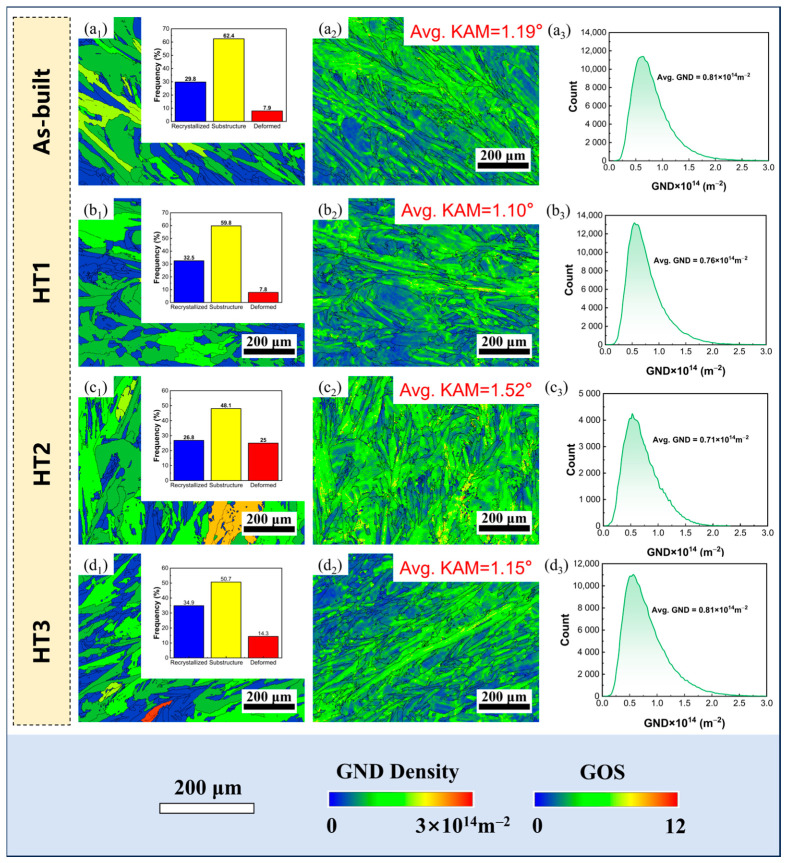
EBSD analysis maps of L-PBF C276 samples after heat treatment: (**a_1_**–**d_1_**) recrystallization distribution maps, (**a_2_**–**d_2_**) KAM distribution maps, and (**a_3_**–**d_3_**) GND density distribution maps.

Further supplementary EBSD analysis reveals significant differences in the degree of recrystallization and local lattice distortion across the various heat treatment conditions (Figure 9(a_1_–d_1_)). The proportions of recrystallized structures in the as-deposited, HT1, HT2, and HT3 states were 29.8%, 32.5%, 26.8%, and 34.9%, respectively. The proportions of substructures were 62.4%, 59.8%, 48.1%, and 50.7%, respectively. The proportions of deformed structures were 7.9%, 7.8%, 25.0%, and 14.3%, respectively. The as-deposited sample exhibited a high proportion of substructures, indicating that a significant amount of subgrain structure, formed through rapid solidification and multiple thermal cycles, was retained in the as-deposited state. The proportion of recrystallized structures in HT1 was slightly higher than that in the as-deposited state, while the proportion of deformed structures remained at a consistently low level. This suggests that low-temperature two-stage aging facilitates a certain degree of recovery and structural stabilization. HT2 displayed the highest proportion of deformed structures, indicating that strong local misorientations and characteristics of non-uniform distortion remained present within its interior. HT3 exhibited the highest proportion of recrystallized structures, demonstrating that the 870 °C + 620 °C two-stage heat treatment is more conducive to structural homogenization and grain boundary transformation.

The KAM (Kernel Average Misorientation) and GND (Geometrically Necessary Dislocations) results further reflect the levels of dislocation storage and local distortion following heat treatment (Figure 9(a_2_–d_3_)). The average KAM values for the as-deposited, HT1, HT2, and HT3 states were 1.19°, 1.10°, 1.52°, and 1.15°, respectively. The higher KAM value observed in the as-deposited sample indicates the presence of residual strain and localized misorientation within its microstructure. The KAM values for HT1 and HT3 were lower than that of the as-deposited state, suggesting that the heat treatments effectively relieved localized lattice distortions to some extent. Conversely, HT2 exhibited the highest KAM value, indicating the greatest degree of localized misorientation and the most pronounced concentration of lattice distortions and short-range strains. Meanwhile, the average GND densities for the as-deposited, HT1, HT2, and HT3 states were 0.81 × 10^14^, 0.76 × 10^14^, 0.71 × 10^14^, and 0.81 × 10^14^ m^−2^, respectively. A comprehensive analysis of the IPF, grain boundary statistics, grain size, KAM, and GND results reveals that HT3 performed optimally in terms of attenuating the textural inheritance of the as-deposited state, increasing the proportion of high-angle grain boundaries (HAGBs), and promoting microstructural homogenization. In contrast, HT2 exhibited more distinct characteristics of localized distortion and grain growth.

### 3.2. Mechanical Property Analysis

**Figure 10:**
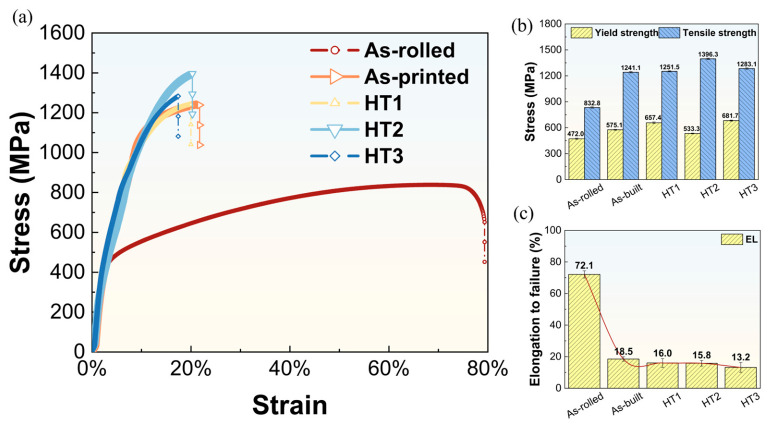
(**a**) Engineering stress–strain curves, (**b**) Statistical results of yield strength and tensile strength, (**c**) Elongation after fracture.

Figure 10a,b present, respectively, the engineering stress–strain curves and the statistical results for the yield strength (YS) and tensile strength (TS) of C276 alloy in various states. It is evident that the L-PBF–deposited samples exhibit significantly higher strength levels compared to the conventional plate samples. The YS and TS of the conventional plate samples were 472.0 MPa and 832.8 MPa, respectively, whereas those of the deposited samples increased to 575.1 MPa and 1241.1 MPa, representing increases of approximately 21.8% and 49.0%, respectively. Concurrently, the elongation at fracture (EL) of the conventional plate samples approached 71.2%, whereas that of the deposited samples was merely about 18.5%. This indicates that the L-PBF process significantly enhanced the material’s strength but simultaneously entailed a marked sacrifice in ductility. This result aligns with the aforementioned microstructural analysis: the cellular/columnar substructures formed during the L-PBF process, along with the elevated dislocation density, residual stresses, and microstructural anisotropy, collectively enhanced the material’s resistance to deformation and load-bearing capacity yet simultaneously constrained the process of uniform plastic deformation, thereby resulting in a distinct reduction in elongation.

Significant differences in mechanical response were observed among samples subjected to different heat treatments. The tensile strengths of HT1, HT2, and HT3 were 1251.5 MPa, 1396.3 MPa, and 1283.1 MPa, respectively, all exceeding that of the as-deposited sample. Among these, HT2 exhibited the highest TS, representing an improvement of approximately 12.5% over the as-deposited state and 67.7% over conventionally processed plate material. In contrast, the yield strengths for the three heat-treated states were 657.4 MPa, 533.3 MPa, and 681.7 MPa. Of these, HT3 demonstrated the highest YS, followed by HT1, while HT2 was relatively lower. Thus, although HT2 possessed the highest ultimate tensile strength, it did not exhibit the highest yield strength. Conversely, HT3 demonstrated the highest initial resistance to yielding but did not achieve the highest ultimate load-bearing capacity. Consequently, HT3 is more effective in enhancing resistance to initial plastic deformation, whereas HT2 is more effective in boosting sustained work-hardening capability and ultimate tensile performance.

It should be noted that the L-PBF process parameters employed in this study represent a stable processing window established through prior optimization. The primary focus of this research lies in investigating the regulation of microstructure and mechanical properties via heat treatment, rather than the systematic optimization of the processing parameters themselves. Nevertheless, from the perspective of microstructure-property relationships, further adjustment of parameters, such as laser power, scanning speed, volumetric energy density, scanning strategy, or substrate preheating conditions, could potentially mitigate residual stress, reduce microstructural anisotropy, and promote a more uniform solidification structure. Such adjustments hold promise for, to some extent, alleviating the characteristic “high strength, low ductility” trade-off, thereby yielding C276 alloy components that exhibit enhanced ductility, albeit with a slight compromise in strength. This issue warrants further systematic investigation in future studies, specifically within the context of the processing parameter window.

**Figure 11:**
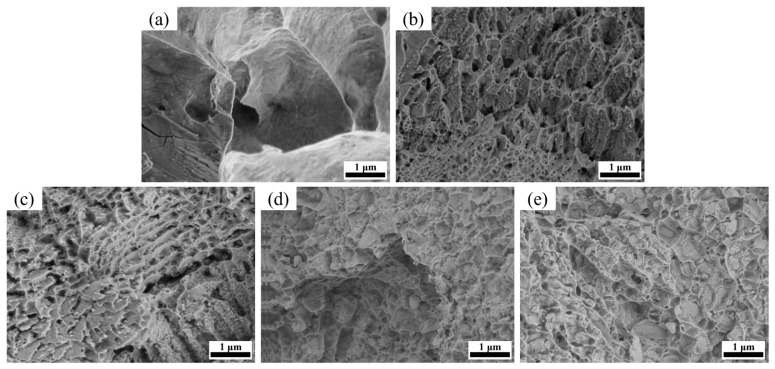
SEM images of tensile fracture surfaces of C276 alloy in different thermal states: (**a**) as-rolled, (**b**) as-printed, (**c**) HT1, (**d**) HT2, and (**e**) HT3.

To further analyze the tensile failure mechanisms of the different samples, the fracture surface morphologies were characterized using SEM, as shown in Figure 11. The results indicate that the fracture surfaces of all samples are predominantly characterized by dimples, suggesting that the overall fracture mechanism is ductile fracture, characterized by the nucleation, growth, and coalescence of microvoids [34]. The conventional plate sample (Figure 11a) exhibits relatively large and deep dimples, accompanied by distinct features of plastic tearing. This suggests that the material underwent substantial plastic deformation prior to fracture, thereby accounting for its highest elongation. The fracture surface of the as-deposited sample (Figure 11b) consists of a multitude of fine, dense dimples. The dimple size is significantly smaller than that of conventional sheet materials, indicating a higher density of void nucleation sites and smaller localized plastic zones. This suggests that although its macroscopic plastic deformation capacity is markedly lower than that of conventional sheets, the fracture process remains predominantly governed by ductile void coalescence.

Following heat treatment, the fracture surface morphologies exhibited varying degrees of modification. The HT1 specimen (Figure 11c) continued to be dominated by dimpled fracture. However, localized dimples appeared elongated and shallow, indicating that the material still exhibited certain characteristics of directional plastic deformation when subjected to high stress levels. On the fracture surface of the HT2 specimen (Figure 11d), the distribution of dimples was relatively uniform, with no evidence of extensive areas of abnormally coarsened voids. This suggests that even at a higher strength level, the material maintained a relatively stable process of micro-void evolution, a finding consistent with its favorable balance between strength and plasticity. In the HT3 specimen (Figure 11e), relatively larger dimples and localized regions of void coalescence were observed. The size distribution of the dimples was less uniform than that of HT2, indicating that localized regions were more susceptible to stress concentration and the premature formation of large void-coalescence zones. Consequently, although this specimen possessed the highest yield strength, its ultimate tensile strength and plasticity did not surpass those of HT2. Overall, samples in all heat-treated states exhibited varying degrees of ductility-limited characteristics. However, their dominant fracture mechanism can still be classified as ductile fracture rather than strictly brittle fracture.

Based on a comprehensive analysis of Figure 10 and Figure 11, as well as the previously described microstructural results, it is evident that the extreme cooling rates inherent to the L-PBF process endow the C276 alloy with a strength and hardness significantly superior to those of conventionally rolled plates. However, the concomitant formation of a cellular substructure leads to a reduction in ductility. Through the induction of μ-phase precipitation and microstructural reconstruction, the applied heat treatment processes successfully optimized the strength–ductility balance of the material. Notably, the HT3 specimen exhibited the highest initial yield strength, a result primarily attributable to fine-grain strengthening, a high proportion of high-angle grain boundaries, and the potent pinning effect exerted by dispersed μ-phase particles on dislocation motion. The HT2 specimen underwent a more thorough microstructural reconstruction, achieving an optimal balance regarding the size and distribution of precipitated μ-phase particles. This, combined with the recovery of the substructure and the elimination of localized lattice distortions, significantly enhanced the material’s capacity for sustained work-hardening and its ultimate load-bearing capability. Consequently, while maintaining an elongation of nearly 20%, the HT2 specimen achieved the highest tensile strength and exhibited more uniform dimpled fracture characteristics, thereby demonstrating the most superior overall mechanical properties. Therefore, a more accurate description of L-PBF–fabricated and heat-treated C276 alloy would be that it exhibits “restricted ductility while retaining toughness-dominated fracture characteristics,” rather than simply classifying it as a brittle material.

### 3.3. Electrochemical Corrosion Analysis

**Figure 12:**
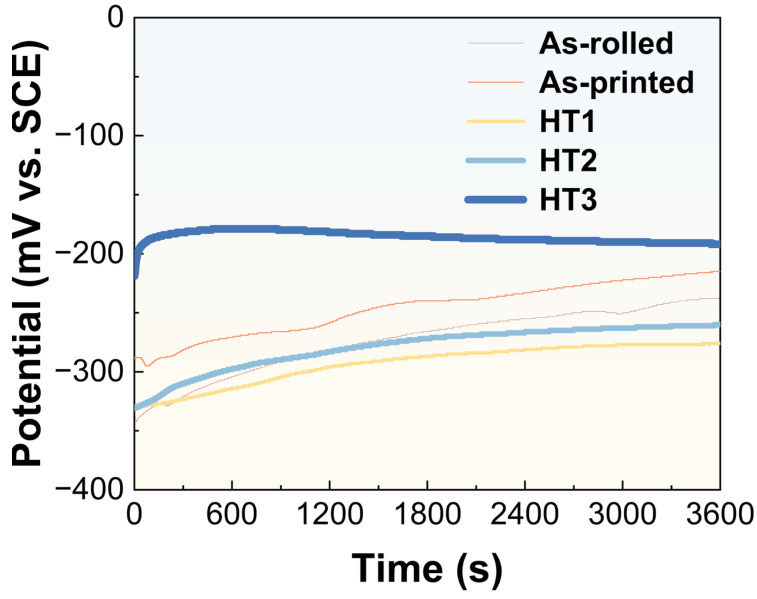
Open circuit potential curves of C276 alloy in different states in a 3.5 wt.% NaCl solution. Potentials are given in mV vs. SCE.

When C276 alloy is widely deployed and operated in marine environments, such as within gas turbines, corrosion emerges as its primary mode of failure due to the influence of humidity and the surrounding environmental media [6]. To evaluate the corrosion resistance of C276 alloy in various heat-treated states within a marine environment, open-circuit potential (OCP), potentiodynamic polarization, and electrochemical impedance spectroscopy (EIS) tests were conducted using a 3.5 wt.% NaCl solution. As illustrated in Figure 12, the potential of all samples gradually stabilized within 3600 s, indicating that the test system successfully attained a relatively stable electrochemical state. With the exception of sample HT3, the OCP values of the remaining samples exhibited a trend characterized by an initial positive shift followed by stabilization. This suggests that during the early stages of immersion, surface active dissolution and the formation of a passive film occur concurrently, eventually leading to the establishment of a dynamic equilibrium. Notably, sample HT3 consistently maintained the most positive open-circuit potential, exhibiting only a slight negative shift during the steady-state phase. This observation suggests that HT3 possesses a strong propensity for initial surface passivation. However, since OCP merely reflects the thermodynamic tendency toward corrosion, it cannot, on its own, serve as a definitive indicator of actual corrosion resistance.

**Figure 13:**
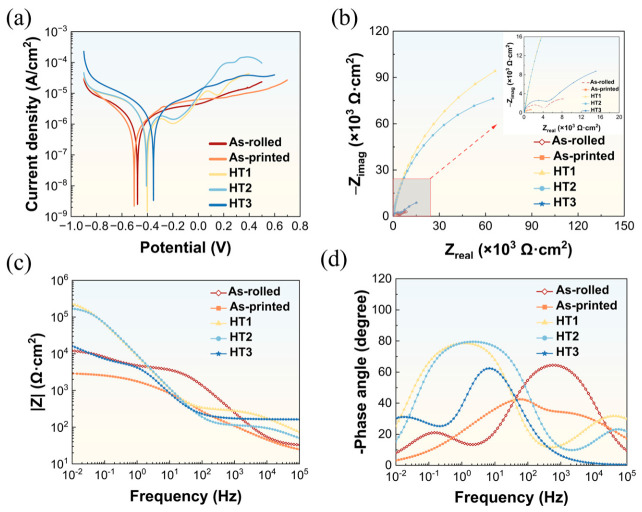
Electrochemical test results of C276 alloy in different states in a 3.5 wt.% NaCl solution: (**a**) polarization curves, (**b**) Nyquist plots, (**c**) Bode impedance modulus plots, and (**d**) Bode phase angle plots.

Figure 13a and Table 7 present the polarization curves and corresponding fitting parameters. The *E_corr_* values for the conventional plate, as-deposited state, HT1, HT2, and HT3 were −0.4795, −0.5090, −0.4038, −0.4165, and −0.3559 V _(vs_. _SCE)_, respectively. Among these, HT3 exhibited the most positive *E_corr_*, while HT1 and HT2 also demonstrated significantly higher values compared to the conventional plate and the as-deposited state. Here, *E_corr_* primarily reflects the thermodynamic tendency of the corrosion reaction and, on its own, does not represent the actual corrosion rate or the stability of the passivation film. However, the *I_corr_* values varied significantly among the different samples. Specifically, HT1 exhibited the lowest *I_corr_* (9.214 × 10^−7^ A/cm**^2^**), followed by HT2 (1.113 × 10^−6^ A/cm**^2^**). Although HT3 possessed the most positive *E_corr_*, its *I_corr_* was notably higher than that of HT1 and HT2, indicating that *E_corr_* and *I_corr_* do not correlate perfectly within this system. Compared to the as-deposited state, the *I_corr_* values of HT1, HT2, and HT3 decreased by approximately 57.3%, 48.4%, and 26.5%, respectively, demonstrating that the heat treatments generally enhanced corrosion resistance, with HT1 yielding the optimal result. Furthermore, as illustrated in Figure 13a, HT1 and HT2 exhibited more pronounced passivation behavior over a relatively wide potential range, whereas the current response of HT3 within the passivation region displayed more significant fluctuations, suggesting that the stability of its passive film is inferior to that of HT1 and HT2.

The shape of the capacitive arc reflects the mechanism of charge transfer. Specifically, the radius of curvature of the capacitive arc is influenced by mass transport and charge transfer within the corrosion product layer on the specimen surface, indicating that the magnitude of the charge transfer resistance can be characterized by the radius of curvature of the capacitive arc [35]. The EIS results further validate this observation. As shown in Figure 13b, the radii of the capacitive arcs in the Nyquist plots follow the order HT1 > HT2 > HT3 > conventional plate > as-deposited state, indicating that the interfacial charge transfer resistance is significantly higher for HT1 and HT2. In the low-frequency region of Figure 13c, the impedance modulus |Z| similarly exhibits the highest values for HT1 and HT2, followed by HT3, with the as-deposited state showing the lowest values. Notably, Figure 13d reveals that HT1 and HT2 exhibit higher and broader phase angle peaks in the low-to-mid frequency range, suggesting that their surface passivation films possess exceptional compactness and that their interfacial capacitive response approaches an ideal state. The underlying reason is that the HT1 treatment achieved elemental homogenization without inducing the precipitation of the μ-phase. This ensured a high degree of chemical homogeneity within the matrix, thereby facilitating the formation of the most complete and continuous protective passivation film. In contrast, the phase angle peak for HT3 is significantly diminished, further corroborating that the excessive precipitation of the μ-phase disrupted the uniformity of the film layer, leading to a localized degradation in the overall barrier performance of the passivation film.

The equivalent circuit fitting results presented in Table 8 are consistent with the aforementioned analysis. For all samples, the value of R_2_ was significantly higher than that of R_1_, indicating that corrosion resistance is primarily governed by the inner barrier layer. The R_2_ values for samples HT1, HT2, and HT3 were 2.836 × 10^5^, 1.842 × 10^5^, and 3.282 × 10^4^ Ω·cm**^2^**, respectively. These values are markedly higher than those of the conventional sheet material and the as-deposited state, demonstrating that heat treatment significantly enhances the barrier capability of the passive film, with HT1 exhibiting the best performance, followed by HT2. Concurrently, HT1 displayed the lowest constant phase element parameter (Q_2_) and the highest dispersion exponent (n_2_, approaching 1.0), suggesting that its passive film possesses an extremely low density of point defects and a physical structure closely approximating that of an ideal capacitor. In contrast, HT3 exhibited a significant increase in Q_2_ and a decrease in n_2_. This physically reveals that the μ-phase precipitates located at specific sites induced local instability and point defect accumulation within the passive film, thereby substantially amplifying the microscopic heterogeneity of the film layer. It should be noted that *E_corr_* reflects the thermodynamic tendency of corrosion, whereas *I_corr_* and EIS parameters primarily reflect corrosion kinetics and the barrier properties of the film layer. Consequently, *E_corr_* does not necessarily vary in perfect synchrony with *I_corr_* and *R*_2_.

### 3.4. Corrosion Mechanism

**Figure 14:**
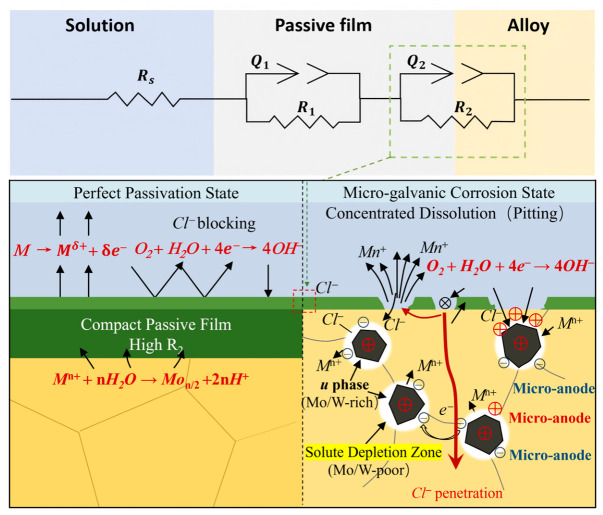
Fitted equivalent circuit and schematic diagram of the sample in a 3.5 wt.% NaCl solution.

Since the Bode phase angle curves for all samples exhibit two primary time constants, this indicates that the interfacial corrosion process can be differentiated into the response of the outer porous film/solution interface and the response of the inner dense barrier film. Consequently, the R_s_(Q_1_R_1_) (Q_2_R_2_) equivalent circuit was employed in this study for fitting purposes. Figure 14 further illustrates the physical model of electrochemical corrosion for the C276 alloy in a NaCl solution. In the equivalent circuit, Rs represents the solution resistance. The Q_1_ to R_1_ pair corresponds to the response of the outer porous corrosion product layer or the layer/electrolyte interface. The Q_2_ to R_2_ pair corresponds the response of the inner dense passive film or the metal/film interface. The fitted χ^2^ values are expressed in scientific notation. Except for HT3, the χ^2^ values of the other samples are mainly in the order of 10^−5^ to 10^−4^, indicating that the fitting results are generally reliable. The higher χ^2^ value of HT3 reflects the greater complexity of its interfacial corrosion process. The significant increase in R_2_ indicates an enhanced protective capability of the inner barrier layer, which is the primary reason for the improved corrosion resistance of HT1 and HT2.

For the HT1 sample, which exhibited no significant precipitation (left side of Figure 14), the matrix maintained a high degree of chemical compositional homogeneity. This uniform microstructure facilitates the homogeneous hydrolysis of metal cations (M^n+^) at the matrix/solution interface (M^n+^ + nH_2_O → MO_n/2_ + 2nH^+^), thereby promoting widespread nucleation across the surface and the growth of a highly dense, continuous, passive protective film. This flawless physical barrier effectively blocks the penetration of aggressive chloride ions (Cl^−^), thereby providing a fundamental physical explanation for why the HT1 sample exhibits an exceptionally high R_2_ value and an extremely low *I_corr_*.

Conversely, for the HT2 and HT3 samples subjected to high-temperature aging (right side of Figure 14), the corrosion mechanism involves micro-galvanic corrosion. As Mo- and W-rich μ phases precipitate extensively along grain boundaries or subgrain boundaries, their nucleation and growth processes severely deplete the surrounding regions of passivating elements, resulting in the formation of distinct solute depletion zones encircling the μ phases. Within a conductive electrolyte solution, the more electropositive μ phases act as micro-cathodes (+), primarily facilitating the oxygen depolarization reduction reaction (O_2_ + 2H_2_O + 4e^−^ → 4OH^−^). Conversely, the more electronegative depletion zones function as micro-anodes (−), undergoing accelerated anodic dissolution of the metal (M → M^n+^ + ne^−^).

This intense localized micro-galvanic effect not only hinders the continuous formation of a dense passivation film but also leads to localized breakdown of the film situated directly above the depletion zone. Chloride ions (Cl^−^) present in the solution preferentially penetrate the film through these microscopic defects, directly attacking the underlying depletion zone and initiating pitting corrosion. This micro-galvanic damage model, predicated on the precipitation of the μ-phase, perfectly corroborates the phenomenon of drastic current fluctuations observed in the polarization curves of the HT3 sample. Furthermore, it provides a mechanistic explanation for why the parameter reflecting film defects (Q_2_) in the equivalent circuit rises significantly, while the resistance of the barrier layer (R_2_) drops sharply.

### 3.5. Analysis of Friction and Wear Tests

**Figure 15:**
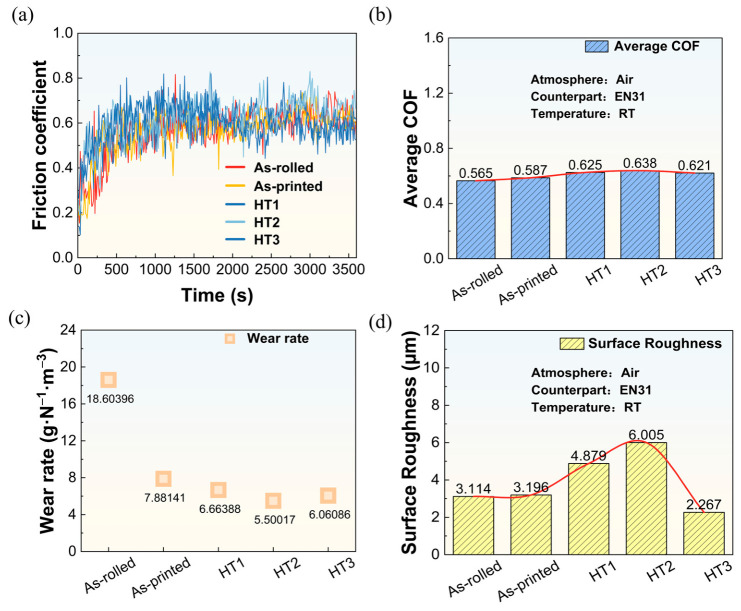
(**a**) Variation of CoF with sliding time, (**b**) average CoF of different samples, (**c**) wear rate and (**d**) surface roughness of different samples after wear.

In dry sliding friction systems, the interfacial coefficient of friction (CoF) exhibits a strong dependence on operating conditions. It typically decreases with increasing sliding velocity or normal load and is significantly influenced by the material of the mating counterpart [36]. Under dry friction conditions in an ambient air environment at room temperature, the friction coefficients of all samples stabilized after undergoing a brief run-in period, indicating that the initial stage of friction primarily corresponds to the bedding-in of contact surfaces and the generation of wear debris. As indicated by Figure 15a and combined with the experiments in this work, the friction coefficients of the various samples typically undergo significant fluctuations during the initial approximately 300 to 500 s of the test, after which they gradually enter a relatively stable phase. This suggests that the interface has essentially completed its natural run-in process during this period. As shown in Figure 15a,b, the average CoFs for the conventional plate, as-deposited state, HT1, HT2, and HT3 samples were 0.565, 0.587, 0.625, 0.638, and 0.621, respectively, with HT2 exhibiting the highest value and the conventional plate the lowest. However, the friction coefficient did not demonstrate a simple, direct correlation with wear resistance. As illustrated in Figure 15c, the wear rates for the respective samples were 18.60396, 7.88141, 6.66388, 5.50017, and 6.06086 g·N^−1^·m^−3^. Compared to the conventional plate, the wear rates of the as-deposited, HT1, HT2, and HT3 samples were reduced by approximately 57.6%, 64.2%, 70.4%, and 67.4%, respectively. This indicates that the L-PBF forming process itself significantly enhanced the alloy’s wear resistance, while subsequent heat treatments further optimized its resistance to damage. Notably, HT2 exhibited the lowest wear rate, suggesting that within this system, the material’s true wear resistance is primarily governed by the load-bearing capacity of the surface matrix, the extent of interfacial material transfer, and the stability of the mechanically mixed layer (MML) rather than being solely determined by the macroscopic friction coefficient. Therefore, if frictional behavior is evaluated solely based on the average coefficient of friction (CoF), traditional sheet samples exhibit the lowest friction coefficient. However, if evaluated from the perspective of comprehensive wear resistance, a more critical factor in engineering applications, HT2 demonstrates the optimal tribological behavior under room-temperature dry sliding conditions.

**Figure 16:**
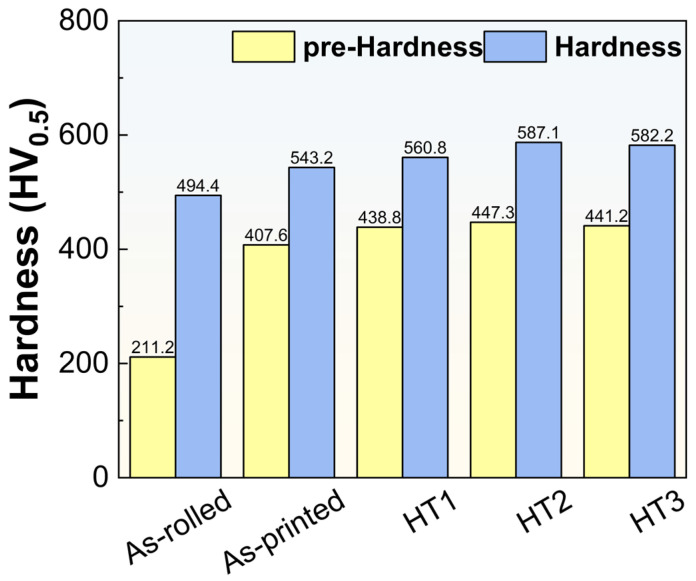
Comparison of microhardness of different samples before and after wear.

Figure 15d shows that following wear testing, the surface roughness values were 3.114, 3.196, 4.879, 6.005, and 2.267 μm, with HT3 exhibiting the lowest value and HT2 the highest. However, the trend in surface roughness does not correlate with the wear rate, indicating that surface roughness alone cannot serve as a sole criterion for evaluating wear resistance. As illustrated in Figure 16, the hardness of all samples increased significantly after wear testing. Specifically, the traditional plate, as-deposited state, HT1, HT2, and HT3 samples saw their hardness values rise from 211.2, 407.6, 438.8, 447.3, and 441.2 HV to 494.4, 543.2, 560.8, 587.1, and 582.2 HV, respectively. This demonstrates that significant work-hardening occurred in all samples during the friction process. Notably, HT2 and HT3 exhibited the highest post-wear hardness values, a result attributed to the strong synergistic strengthening effect arising from the high-density dislocations induced by severe plastic deformation and the dispersively distributed hard μ-phase particles within the matrix.

**Figure 17:**
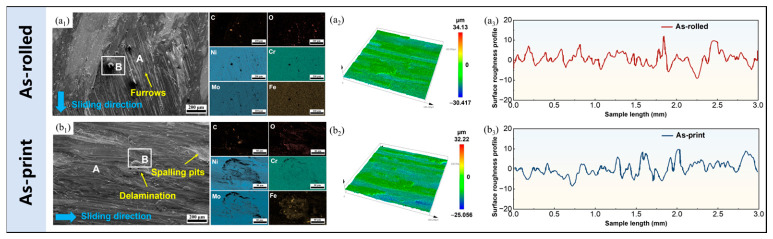
SEM images of wear tracks for the conventional plate and as-deposited state (**a_1_**,**b_1_**), along with the elemental composition (at.%) of the wear regions; (**a_2_**,**b_2_**) overall profiles after wear testing; (**a_3_**,**b_3_**) wear depths after wear testing.

**Figure 18:**
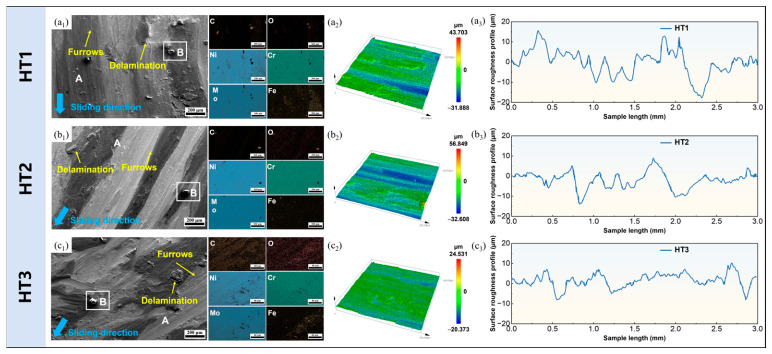
(**a_1_**–**c_1_**) SEM images of wear tracks for HT1, HT2, and HT3, and the elemental composition (at.%) of the wear regions; (**a_2_**–**c_2_**) overall surface profiles after wear testing; (**a_3_**–**c_3_**) wear depths after wear testing.

The morphology of the wear tracks further elucidated the wear mechanisms of the different samples. Figure 17 and Figure 18 reveal that the surfaces of the conventional plate and the as-deposited sample exhibited distinct ploughing grooves, spalling pits, and delamination, indicating that they were more susceptible to abrasive wear and fatigue spalling under cyclic shear stress. In contrast, the wear track surfaces of samples HT1 and HT2 appeared smoother, with shallower grooves and smaller areas of delamination, suggesting that the load-bearing capacity of their surface layers was significantly enhanced following heat treatment. Although the overall degree of damage in sample HT3 was lower than that of the conventional plate, the presence of relatively large wear debris and localized delamination was still evident, indicating that its interfacial stability was inferior to that of HT2. Correlating these observations with the EDS results presented in Table 9, it was determined that Region A primarily corresponds to the matrix or the transfer layer. It is characterized by high concentrations of Ni, Cr, and Mo, alongside a certain amount of Fe, which signifies material transfer from the EN31 counterpart. Notably, the Fe content in Region A of sample HT2 was merely 2.55 at.%, a figure significantly lower than the 5.91 at.% observed in the conventional plate and the 6.58 at.% in HT3, suggesting that adhesive tearing at the interface and material transfer from the counterpart were less severe in HT2. Region B, conversely, generally exhibited elevated concentrations of C and O, indicating that it consists primarily of a mechanically mixed layer (MML) formed by the compaction of wear debris, as well as localized oxidized wear debris. However, based solely on EDS analysis, these constituents cannot be definitively identified as specific oxide phases. Synthesizing the findings regarding wear track morphology and elemental composition, it is concluded that the dominant wear mechanisms for all samples under the specified operating conditions were adhesive wear and abrasive wear, accompanied by minor oxidative wear. Among the samples, HT2, benefiting from the optimal subsurface support provided by its μ-phase, maintained the most stable MML interface, thereby achieving the lowest wear rate.

**Figure 19:**
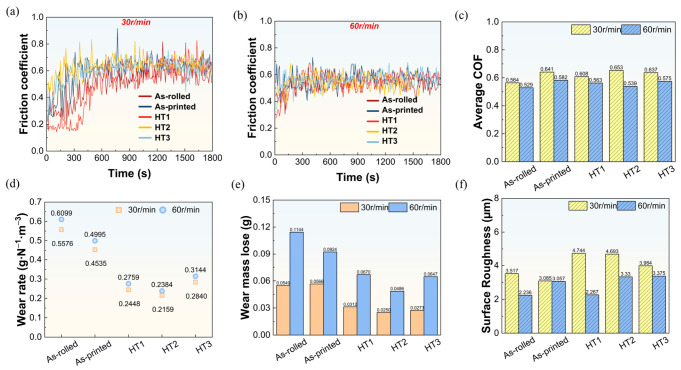
The heat-treated alloy at rotational speeds (**a**) 30 r/min and (**b**) 60 r/min; (**c**) average friction coefficient; (**d**) wear rate; (**e**) mass loss; (**f**) surface roughness after wear.

A further examination of the influence of rotational speed on wear rate reveals that as the speed increases from 30 r/min to 60 r/min, the average friction coefficient for all samples decreases, as shown in Figure 19a–c. However, their wear rates all exhibit an upward trend. As shown in Figure 19d,e, the wear rates for the traditional plate, as-deposited state, HT1, HT2, and HT3 at 30 r/min were 0.5576, 0.4535, 0.2448, 0.2159, and 0.2840 g·N^−1^·m^−3^, respectively. At 60 r/min, these values rose to 0.6099, 0.4995, 0.2759, 0.2384, and 0.3144 g·N^−1^·m^−3^, corresponding to increases of approximately 9.38%, 10.14%, 12.70%, 10.42%, and 10.70%, respectively. Notably, HT2 maintained the lowest wear rate at both rotational speeds, and its absolute increase, at only 0.0225 g·N^−1^·m^−3^, was the smallest among all samples, thereby demonstrating superior interfacial stability under high-speed conditions. In other words, under both constant-speed conditions of 30 r/min and 60 r/min, HT2 consistently demonstrated the optimal comprehensive wear resistance. Conversely, although the traditional plate exhibited a lower average coefficient of friction (CoF), it recorded the highest wear rate. Therefore, it cannot be considered to possess the optimal tribological behavior.

As shown in Figure 19f, with increasing rotational speed, the surface roughness of the wear tracks on certain samples actually decreases: the traditional plate material dropped from 3.517 to 2.236 μm, HT1 from 4.744 to 2.267 μm, and HT2 from 4.693 to 3.330 μm. This seemingly contradictory phenomenon, where the surface becomes smoother while wear volume increases, stems from the sharply elevated interfacial flash temperatures generated at high rotational speeds. The accumulation of frictional heat renders wear debris more susceptible to softening, oxidation, and compaction, thereby smoothing out the surface topography (reducing both roughness and the coefficient of friction). Concurrently, however, this thermal softening effect diminishes the yield strength of the subsurface matrix, thereby exacerbating overall material yielding and removal [37].

**Figure 20:**
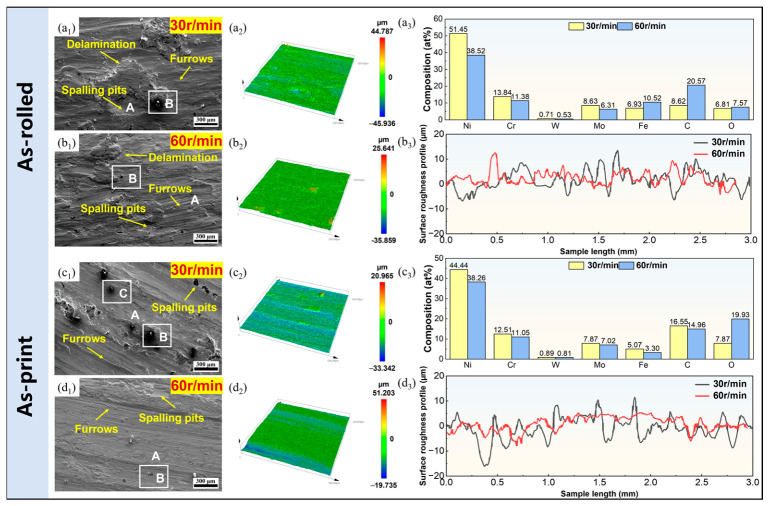
Characterization of wear tracks on conventional plates and as-deposited samples at different rotational speeds: (**a_1_**,**a_2_**) Conventional plate, 30 r/min. (**b_1_**,**b_2_**) Conventional plate, 60 r/min. (**c_1_**,**c_2_**) As-deposited state, 30 r/min. (**d_1_**,**d_2_**) As-deposited state, 60 r/min. Herein, 1 represents the SEM image of the wear track, and 2 represents the overall profile after wear. (**a_3_**,**c_3_**) show the elemental composition (at.%) of the wear track region, while (**b_3_**,**d_3_**) show the wear track profile curves.

**Figure 21:**
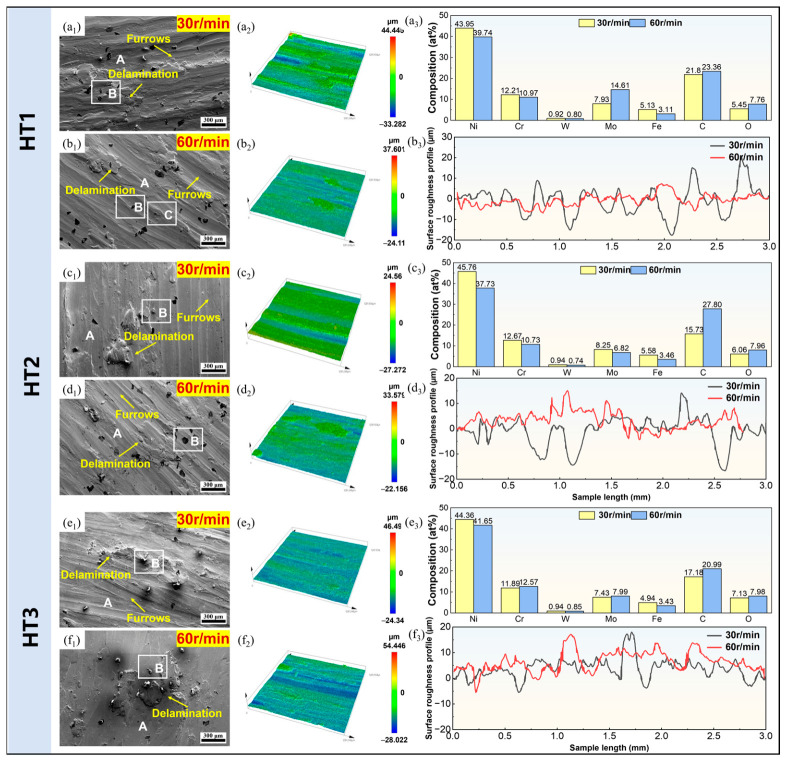
Characterization of wear tracks on heat-treated samples at different rotational speeds: (**a_1_**,**a_2_**) HT1, 30 r/min. (**b_1_**,**b_2_**) HT1, 60 r/min. (**c_1_**,**c_2_**) HT2, 30 r/min. (**d_1_**,**d_2_**) HT2, 60 r/min. (**e_1_**,**e_2_**) HT3, 30 r/min. (**f_1_**,**f_2_**) HT3, 60 r/min. Here, 1 represents the SEM image of the wear track, and 2 represents the overall profile map after wear. (**a_3_**,**c_3_**,**e_3_**) show the elemental composition (at.%) of the wear track region, and (**b_3_**,**d_3_**,**f_3_**) show the wear track profile curves.

The wear depth does not increase in direct proportion to the rotational speed. This may be attributed to the influence of the “groove effect” on the abrasive particles generated during the wear process [37]. Figure 20 and Figure 21 indicate that as the rotational speed increases, the prevalence of spalling pits on the surfaces of the conventional plate, as-deposited, and heat-treated samples generally decreases, while signs of delamination and adhesion become more pronounced. This suggests that at higher rotational speeds, frictional heat intensifies the compaction and localized re-attachment of wear debris, thereby shifting the dominant wear mechanism from abrasive plowing toward a synergistic combination of adhesion and delamination. The conventional plate samples exhibited distinct plowing grooves, spalling pits, and delamination at both rotational speeds, indicating that their wear behavior remained predominantly characterized by abrasive wear and fatigue spalling. The extent of surface damage was reduced in the as-deposited samples. However, a significant number of spalling pits and plowing grooves remained evident. At a rotational speed of 60 r/min, the surfaces of the HT1 and HT2 samples appeared smoother, with an increase in delamination zones and a reduction in spalling pits. Notably, HT2 maintained shallower plowing grooves and fewer instances of severe spalling, thereby demonstrating a more stable interfacial state. In contrast, the HT3 samples exhibited more pronounced delamination and localized adhesive accumulation at high rotational speeds, suggesting a greater sensitivity to increases in sliding velocity.

This indicates that under the influence of friction-induced heat, the dominant wear mechanism gradually evolves from abrasive plowing toward a synergistic adhesion-delamination mechanism. Under extreme high-speed operating conditions, both HT1 and HT2 manage to maintain relatively smooth surfaces. Notably, HT2 exhibits the shallowest spalling pits. This conclusively demonstrates that the μ-phase within HT2, characterized by its moderate size and uniform distribution, exhibits exceptional thermal stability and resistance to softening within high-temperature tribological environments. Consequently, it provides continuous, robust support to the overlying MML layer, thereby slowing the propagation of subsurface fatigue cracks.

EDS results (see Table 10 and Table 11) further indicate that at high rotational speeds, Region B remains dominated by a carbon-rich mechanically mixed layer. However, the oxygen content did not increase concomitantly across all samples. In summary, the heat-treatment-induced precipitation of the μ-phase fundamentally restructured the micromechanical response of the L-PBF C276 alloy. By virtue of its high-hardness matrix, extremely low tendency for material adhesion, and superior load-bearing capacity within the mechanically mixed layer, sample HT2 demonstrated the optimal overall wear resistance across various wear conditions.

### 3.6. Analysis of Friction Mechanisms

**Figure 22:**
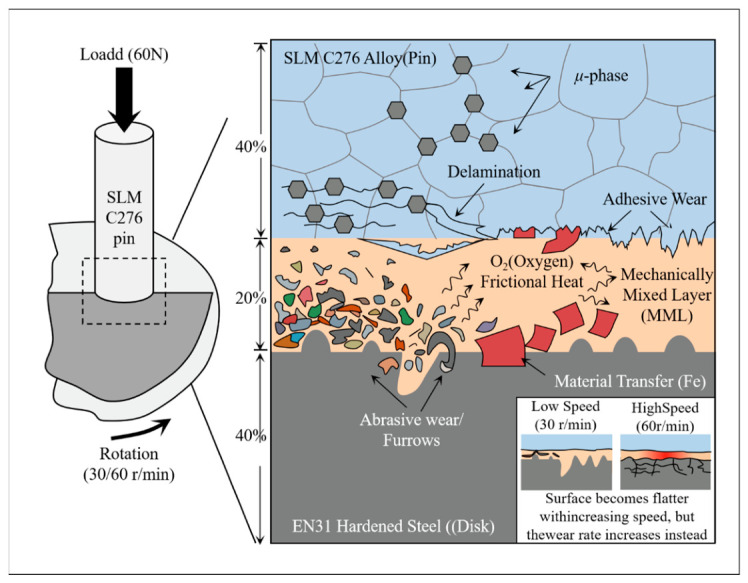
Physical model of the friction and wear machine.

Based on a comprehensive analysis of micro-morphology, compositional evolution, and macro-tribological data, Figure 22 establishes a physical model of the wear mechanisms for L-PBF C276 alloy under dry sliding friction conditions. Under the combined action of normal force and alternating shear forces, the rise in flash temperature at the friction interface promotes the reaction of detached wear debris with ambient oxygen. Subjected to repeated rolling and compaction, these oxidation products, along with iron elements transferred from the mating counterpart (EN31), consolidate at the interface to form a dense mechanically mixed layer (MML). Overall, the dominant wear mechanisms within this system are adhesive wear and abrasive wear, accompanied by concomitant fatigue delamination and oxidative wear.

The Mo/W-rich μ-phase, induced by heat treatment, fundamentally reconstructs the micromechanical response of the matrix. On one hand, the dispersively distributed, high-hardness μ-phase particles act as a robust load-bearing skeleton. They effectively resist indentation and plowing (abrasive wear) by the micro-asperities of the mating counterpart while providing strong geometric support to the MML layer. This significantly reduces the extent of interfacial adhesive tearing and material transfer, the fundamental reason behind the substantial improvement in the wear resistance of the heat-treated alloys (particularly HT2). On the other hand, owing to the significant mismatch in elastic modulus between the hard μ-phase and the ductile, Ni-rich matrix, intense stress concentrations readily develop at the μ-phase interfaces under extreme shear stress. This stress concentration promotes the nucleation of subsurface cracks, which propagate along the boundaries of the precipitated phases and eventually coalesce, leading to the fatigue-induced delamination and spalling of the overlying MML and portions of the matrix material.

Changes in sliding speed further induced a dynamic transition in the wear mechanisms. At low speeds (30 r/min), the accumulation of interfacial frictional heat was relatively slow. Wear was predominantly governed by mechanical cutting, manifesting as characteristic microscopic furrows and localized adhesive material transfer. Under these conditions, the mechanical support provided by the μ-phase played an absolutely dominant role, and the crack propagation rate remained relatively slow.

However, at high rotational speeds (60 r/min), the sharply elevated frictional heat generated at the interface gives rise to a significant synergistic effect. On one hand, the high temperature accelerates oxidation kinetics, promoting the further plasticization and compaction of the MML layer. This effectively smooths out surface micro-irregularities, leading to an anomalous reduction in both the macroscopic coefficient of friction (CoF) and surface roughness. On the other hand, however, the subsurface thermal softening induced by the high temperature weakens the substrate’s yield strength, thereby greatly accelerating the propagation rate of fatigue cracks within the stress concentration zones surrounding the μ-phase. Consequently, the wear mechanism rapidly shifts from one dominated by micro-ploughing to a synergistic mode of large-scale adhesive-delamination damage, ultimately resulting in a significant increase in both material removal volume and actual wear rate at high rotational speeds.

### 3.7. Comparative Analysis with Existing Studies

Compared to the existing literature, the results of this study generally align with established principles regarding the post-heat-treatment behavior of additively manufactured C276 alloys, yet they also highlight new areas of focus. First, existing studies on WAAM/DED processes generally posit [25,26] that high-temperature post-heat treatments can promote recrystallization, attenuate the “inherited” characteristics of directional solidification, and restructure the distribution of secondary phases. This thereby significantly alters the balance between strength and ductility. The microstructural homogenization observed in HT2 and HT3 in this study, along with the attendant modifications to grain boundary characteristics, is consistent with these established principles. Second, regarding the corrosion behavior of C276, previous studies have indicated [27] that during the aging process, Mo/W-rich precipitates and the surrounding solute-depleted zones can compromise the stability of the passive film. This study further demonstrates that within the context of L-PBF–fabricated C276 alloys, this specific microstructural effect results in the localized corrosion resistance of HT2 and HT3 being inferior to that of HT1, in which no significant precipitation occurred. Third, concerning wear behavior, the existing studies on surface modification or coating of C276 alloys have shown [38,39] that grain refinement and the structural support provided by hard phases can effectively reduce material removal rates. This study further reveals that within the L-PBF–fabricated C276 alloy system, a moderate amount of μ-phase precipitation not only enhances the load-bearing capacity of the matrix but also stabilizes the mechanically mixed layer (MML). Consequently, HT2 exhibits the lowest wear rate across a range of rotational speeds. Thus, building upon the existing understanding of the “heat treatment–microstructure–mechanical properties” relationship, this study further establishes a direct correlation between “heat treatment, microstructural reconstruction, and integrated corrosion/wear response,” thereby enriching our understanding of the principles governing performance optimization for L-PBF–fabricated C276 alloys in complex service environments.

## 4. Conclusions

C276 alloy was fabricated using L-PBF technology. Three distinct heat treatment processes were selected, and their effects on the surface morphology, microstructure, and microhardness of the alloy were comprehensively analyzed. Subsequently, electrochemical corrosion tests and friction and wear tests were conducted. Based on the aforementioned analyses, the following conclusions were drawn:

(1) The as-deposited C276 alloy fabricated via L-PBF exhibited characteristic fish-scale-like melt pool boundaries, a cellular/columnar non-equilibrium solidification structure, and pronounced microstructural anisotropy. Heat treatment significantly drove the redistribution of solute atoms. HT1 (720 °C) achieved elemental homogenization primarily through substructure stabilization, with no distinct precipitate phases observed. Conversely, high-temperature heat treatments (HT2 and HT3) induced the dispersed precipitation of Mo- and W-rich topologically close-packed (TCP) phases, specifically the μ-phase, along grain boundaries and cell walls. Following a secondary aging treatment in HT3, the volume fraction of the precipitated μ-phase increased further.
(2) L-PBF processing significantly enhanced the strength and hardness of the C276 alloy. Heat treatment further optimized the balance between strength and ductility. Specifically, HT2 (870 °C × 8 h, furnace cooling) exhibited the most comprehensive mechanical properties, achieving a tensile strength (TS) of 1396.3 MPa, approximately 1.68 times that of conventionally produced plates. The significant increase in hardness observed in HT2 and HT3 is attributed to the pinning effect of hard μ-phase precipitates, whereas the superior initial yield resistance demonstrated by HT3 stems from the strong impediment to dislocation motion caused by a finer distribution of precipitate phases. Furthermore, the results of this study indicate that while heat treatment effectively optimizes the strength–ductility balance of L-PBF C276 alloys, integrating this process with the optimization of L-PBF processing parameters holds the potential to further enhance ductility at the cost of a moderate sacrifice in strength, thereby achieving superior overall service performance.
(3) Heat treatment generally improved the alloy’s corrosion resistance in a 3.5 wt.% NaCl solution. HT1 exhibited the optimal corrosion resistance, characterized by the formation of the densest and most intact passive film, because it avoided the precipitation of the μ-phase and achieved a highly uniform chemical composition within the matrix. In contrast, the precipitation of the μ-phase in HT2 and HT3 led to the formation of solute-depleted zones in the surrounding regions, triggering intense micro-galvanic corrosion effects that resulted in the localized breakdown of the passive film (manifested by a significant decrease in R2 and an increase in Q2). HT3, which contained the highest volume fraction of μ-phase precipitates, exhibited the lowest passive film stability, indicating that over-aging precipitation significantly degrades the material’s localized corrosion resistance.
(4) Under dry sliding friction at room temperature, the dominant wear mechanisms for the C276 alloy were adhesion wear and abrasive wear, accompanied by minor oxidative wear. HT2 demonstrated superior wear resistance, exhibiting the lowest wear rate, approximately 70.4% lower than that of conventional plates, a result attributed to the μ-phase providing robust geometric support to the surface mechanical mixing layer (MML). As the rotational speed increased from 30 r/min to 60 r/min, the rise in interfacial flash temperature promoted the compaction of wear debris (resulting in reduced surface roughness). However, the concomitant thermal softening effect exacerbated material removal. Across all tested rotational speeds, HT2 consistently maintained the lowest wear rate and the most stable interfacial state, thereby demonstrating superior adaptability to varying operating conditions.
(5) Engineering application recommendations: If service conditions prioritize corrosion resistance, HT1 is the more suitable choice for the preferred heat treatment process. Conversely, if a component is simultaneously subjected to mechanical loads and tribological wear, HT2 demonstrates greater potential for engineering application. This is because it combines the highest tensile strength, high hardness, and the lowest wear rate while also exhibiting the most stable interfacial state across various rotational speeds. In comparison, although HT3 achieves a higher degree of microstructural homogenization, its overall performance does not surpass that of HT1 or HT2. Therefore, for C276 alloy components operating in marine environments, where demands for both corrosion resistance and wear resistance coexist, HT2 is the recommended heat treatment state for service.

Overall, the results presented in this paper align with existing research trends regarding how post-heat treatments facilitate microstructural reconstruction and property modulation in C276 alloys. However, this study further extends this scope to provide a systematic evaluation of the synergistic response between corrosion resistance and wear resistance in L-PBF–fabricated C276 alloys, indicating that HT1 is better suited for service scenarios where corrosion resistance is the primary requirement, whereas HT2 is more appropriate for complex operating conditions involving simultaneous exposure to tribological wear and corrosion. 

## Figures and Tables

**Table 1:** Chemical composition of C276 alloy powder (wt.%).

Elements	Ni	Cr	Mo	Mn	Fe	W	Si	O	V	P
wt.%	58.54	15.5	16.1	1.20	2.90	4.60	0.60	0.04	0.50	0.02

**Table 2:** Heat treatment parameters of the samples.

Sample Type	Heat Treatment Processes
As-rolled	/
As-printed	/
HT1	720 °C × 8 h (FC ^1^, 50 °C/h) + 620 °C × 8 h (FC)
HT2	870 °C × 8 h (FC)
HT3	870 °C × 8 h (FC, 50 °C/h) + 620 °C × 8 h (FC)

^1^ Note: FC: furnace cooling.

**Table 3:** Electrochemical corrosion test parameters.

Test	Setting	Parameter
Open circuit potential (OCP)	Time	3600 s
Potentiodynamic polarization (PD)	Sweep range	−1 V to1 V
	Sweep rate	1 mV·s^−1^
Electrochemical impedance spectroscopy (EIS)	Frequency range	10^5^ Hz to 10^−2^ Hz
	Potential amplitude	5 mV

**Table 4:** Chemical composition of EN31 alloy (wt.%).

Elements	Fe	Mn	Cr	Si	Al	Ni	Cu	Mo	S	Ti
wt.%	97.93	0.52	1.16	0.16	0.11	0.02	0.05	0.02	0.01	0.02

**Table 5:** Friction and wear test parameters.

Load (N)	Rotational Speed (r/min)	Duration (h)	Temperature (°C)
60	30 + 60	1	26
60	30	0.5	26
60	60	0.5	26

**Table 6:** Results of EDS point analysis (at.%) of the precipitate phase in region A (Figure 5).

Samples/at.%	Ni	Cr	Fe	Mo	W	Si	C
HT2	40.47	16.84	2.51	22.89	3.06	1.39	11.29
HT3	46.12	17.49	2.98	13.80	2.60	1.45	8.60

**Table 7:** The corrosion potential (*E_corr_*) and corrosion current density (*I_corr_*) of the samples in a 3.5 wt.% NaCl solution.

Samples	*E_corr_* (V _vs_. SCE)	*I_corr_* (A/cm^2^)
As-rolled	−0.4795	1.925 × 10^−6^
As-printed	−0.5090	2.157 × 10^−6^
HT1	−0.4038	9.214 × 10^−7^
HT2	−0.4165	1.113 × 10^−6^
HT3	−0.3559	1.586 × 10^−6^

Note: *E_corr_* is determined from the potentiodynamic polarization curve, while *I_corr_* is obtained by extrapolating from the cathodic Tafel linear region in the vicinity of *E_corr_*. The anodic branch was not utilized for Tafel extrapolation, as it is influenced by the formation of a passivation film, localized film breakdown, and subsequent repassivation behavior.

**Table 8:** Fitted impedance parameters of the samples in 3.5 wt.% NaCl solution.

Samples	R_s_ /(Ω·cm^2^)	Q_1_ /(S·cm^−2^·s^n^)	n_1_	R_1_ /(Ω·cm^2^)	Q_2_ /(S·cm^−2^·s^n^)	n_2_	R_2_ /(Ω·cm^2^)	χ^2^
As-rolled	31.0	3.334 × 10^−6^	0.8148	4101.0	2.141 × 10^−4^	0.7299	9.052 × 10^3^	4.577 × 10^−4^
As-printed	16.3	1.138 × 10^−4^	0.5029	625.6	2.089 × 10^−6^	0.7698	2.457 × 10^3^	3.299 × 10^−4^
HT1	32.5	3.912 × 10^−6^	0.6347	297.0	1.805 × 10^−5^	0.9443	2.836 × 10^5^	8.591 × 10^−5^
HT2	32.6	1.522 × 10^−6^	0.7547	80.0	1.992 × 10^−5^	0.9143	1.842 × 10^5^	1.510 × 10^−4^
HT3	170.0	3.048 × 10^−5^	0.8840	5769.0	4.189 × 10^−4^	0.6811	3.282 × 10^4^	1.601 × 10^−3^

Note: χ^2^ is a dimensionless fitting error parameter, which is expressed in scientific notation in this paper.

**Table 9:** EDS analysis results (at%) for regions A and B, marked on the sample in Figure 17.

Samples	Point	Element Composition/at.%						
		Ni	Cr	W	Mo	Fe	C	O
As-rolled	A	55.74	15.92	0.85	9.90	5.91	5.30	3.98
	B	4.51	4.16	0.50	7.74	0.91	52.33	13.52
As-printed	A	59.73	13.35	1.61	10.01	4.04	2.91	9.27
	B	35.18	13.23	0.93	7.13	33.71	3.93	8.53
HT1	A	51.65	13.37	1.12	9.17	3.75	8.60	7.47
	B	5.71	4.08	0.21	2.99	1.08	42.99	24.43
HT2	A	50.75	13.47	1.52	12.17	2.55	8.54	5.70
	B	17.08	10.16	0.57	5.97	0.79	51.09	3.56
HT3	A	49.24	14.24	1.04	9.32	6.58	9.59	5.83
	B	19.58	0.51	0.18	1.95	0.48	22.06	21.77

**Table 10:** EDS analysis results (at.%) for the marked points A, B, and C on the samples shown in Figure 20 and Figure 21 at a speed of 30 r/min.

Samples	Point	Element Composition/at.%						
		Ni	Cr	W	Mo	Fe	C	O
As-rolled	A	56.96	16.27	0.83	10.10	6.10	4.40	2.94
	B	1.60	1.12	0.02	0.63	2.15	37.36	38.48
As-printed	A	45.17	14.98	0.95	8.76	5.08	15.89	6.13
	B	4.63	1.93	0.14	1.48	0.82	59.54	19.15
	C	0.30	0.60	0	0.04	0.04	2.44	69.70
HT1	A	52.96	12.56	1.21	9.42	4.20	6.04	7.33
	B	5.36	2.38	0.16	1.76	0.63	65.63	17.00
HT2	A	56.02	13.80	1.16	9.11	5.18	3.86	5.07
	B	5.57	3.10	0.20	2.23	0.69	58.11	20.30
HT3	A	55.95	14.57	1.19	9.69	3.08	4.30	5.74
	B	9.39	6.48	0.35	4.16	2.10	62.51	7.95

**Table 11:** EDS analysis results (at.%) for the marked points A, B, and C on the samples shown in Figure 20 and Figure 21 at a speed of 60 r/min.

Samples	Point	Element Composition/at.%						
		Ni	Cr	W	Mo	Fe	C	O
As-rolled	A	57.11	14.93	0.86	9.92	5.65	3.89	4.04
	B	4.66	1.36	0.08	1.30	0.56	60.80	18.82
As-printed	A	38.99	11.90	0.83	7.56	3.50	4.26	29.07
	B	3.91	1.28	0.10	1.21	0.40	64.30	13.38
HT1	A	55.99	16.28	1.18	10.35	4.21	4.41	3.31
	B	5.33	2.18	0.17	1.71	0.32	70.56	10.35
	C	11.11	4.00	0.28	2.75	1.96	9.83	48.14
HT2	A	48.06	11.13	1.21	9.58	3.99	8.40	11.95
	B	17.60	5.99	0.39	3.42	0.88	55.30	8.88
HT3	A	48.30	12.83	0.94	8.18	9.82	12.99	3.36
	B	3.41	1.42	0.10	1.19	0.69	59.54	18.83

## Data Availability

The original contributions presented in this study are included in the article. Further inquiries can be directed to the corresponding authors.

## Abbreviations

The following abbreviations are used in this manuscript:

L-PBF	Laser Powder Bed Fusion
SLM	Selective Laser Melting
SEM	Scanning Electron Microscope
EDS	Energy Dispersive X-ray Spectrometer
EBSD	Electron Backscatter Diffraction
OCP	Open Circuit Potential
EIS	Electrochemical Impedance Spectroscopy
CoF	Coefficient of Friction
MML	Mechanical Mixing Layer
